# Antioxidant, Anti-α-Glucosidase, Antityrosinase, and Anti-Inflammatory Activities of Bioactive Components from *Morus alba*

**DOI:** 10.3390/antiox11112222

**Published:** 2022-11-11

**Authors:** Jui-Hung Hsu, Chang-Syun Yang, Jih-Jung Chen

**Affiliations:** 1 Department of Pharmacy, School of Pharmaceutical Sciences, National Yang Ming Chiao Tung University, Taipei 112304, Taiwan; 2 Department of Medical Research, China Medical University Hospital, China Medical University, Taichung 404332, Taiwan

**Keywords:** *Morus alba*, active components, antioxidant activity, anti-inflammatory activity, anti-α-glucosidase activity, antityrosinase activity, molecular docking

## Abstract

The root bark of *Morus alba* L. (Mori Cortex) is used to treat diuresis and diabetes in Chinese traditional medicine. We evaluated different solvent extracts and bioactive components from the root bark of *Morus alba* L. for their antioxidant, anti-α-glucosidase, antityrosinase, and anti-inflammatory activities. Acetone extract showed potent antioxidant activity, with SC_50_ values of 242.33 ± 15.78 and 129.28 ± 10.53 µg/mL in DPPH and ABTS radical scavenging assays, respectively. Acetone and ethyl acetate extracts exhibited the strongest anti-*α*-glucosidase activity, with IC_50_ values of 3.87 ± 1.95 and 5.80 ± 2.29 μg/mL, respectively. In the antityrosinase assay, the acetone extract showed excellent activity, with an IC_50_ value of 7.95 ± 1.54 μg/mL. In the anti-inflammatory test, ethyl acetate and acetone extracts showed significant anti-nitric oxide (NO) activity, with IC_50_ values of 10.81 ± 1.41 and 12.00 ± 1.32 μg/mL, respectively. The content of the active compounds in the solvent extracts was examined and compared by HPLC analysis. Six active compounds were isolated and evaluated for their antioxidant, anti-*α*-glucosidase, antityrosinase, and anti-inflammatory properties. Morin (**1**) and oxyresveratrol (**3**) exhibited effective antioxidant activities in DPPH and ABTS radical scavenging assays. Additionally, oxyresveratrol (**3**) and kuwanon H (**6**) showed the highest antityrosinase and anti-α-glucosidase activities among all isolates. Morusin (**2**) demonstrated more significant anti-NO and anti-iNOS activities than the positive control, quercetin. Our study suggests that the active extracts and components from root bark of *Morus alba* should be further investigated as promising candidates for the treatment or prevention of oxidative stress-related diseases, hyperglycemia, and pigmentation disorders.

## 1. Introduction

Free radicals, such as superoxide radicals (O_2_^•−^), hydrogen peroxide (H_2_O_2_), hydroxyl radicals (^•^OH), and singlet oxygen (^1^O_2_), are generated by natural biochemical processes in the body. These free radicals are defined as reactive oxygen species (ROS) [1]. If the level of ROS is too high or the capacity of the antioxidant system is decreased, oxidative stress increases, likely damaging lipids, proteins, and DNA. Oxidative stress is associated with inflammation, aging, hyperpigmentation disorders, diabetes, cancer, atherosclerosis, ischemic heart disease, and neurodegenerative disorders [2]. Some synthetic antioxidants, such as butylated hydroxyanisole (BHA) and butylated hydroxytoluene (BHT), are commonly used to combat oxidative stress, but they are thought to cause liver and kidney damage and carcinogenesis [3,4]. Natural antioxidant products may be safer and more effective, so their efficacy in reducing ROS levels compared to synthetic antioxidants is of concern [5].

As mentioned above, oxidative stress is associated with many diseases, such as neurodegenerative disorders, inflammation, hyperpigmentation, and diabetes. Several studies have shown that oxidative stress plays a crucial role in diabetes and its complications [6,7,8]. For example, a previous study showed that oxidative stress-mediated loss of beta-cell function impaired secretory capacity and increased insulin resistance [7]. Another study showed that ROS cause insulin resistance and diabetic complications by inducing proinflammatory macrophages [8].

Diabetes is a chronic metabolic disease classified as type I or type II diabetes. Type II diabetes is caused by insulin resistance or impairment, resulting in abnormally high blood glucose levels [9]. One way to treat diabetes is to inhibit α-glucosidase in the gut, a key enzyme involved in the digestion of carbohydrates, slowing glucose absorption and reducing the rate at which blood sugar increases after meals. Acarbose and miglitol, as competing inhibitors, are examples of known drugs [10,11]. In recent years, many plant components have been found to exhibit *α*-glucosidase-inhibitory activity and have been used in the development of food or lead compounds for the treatment of diabetes, such as flavonoids and phenolic compounds [12].

Hyperpigmentation is a disorder of excessive accumulation of melanin due to upregulation of melanin synthesis and reduced breakdown of melanosomes, including melasma, postinflammatory hyperpigmentation (PIH), age spots, and solar lentigo [13]. Many studies suggest that oxidative stress plays a key role in the production and progression of pigmentation; UV-induced oxidative stress and DNA damage in skin cells initiates cellular signals that promote melanogenesis, and the relationship between oxidative stress and UVA-mediated aggravation of melanogenesis occurs as a result of overproduction of reactive oxidants [14,15,16]. Tyrosinase, a copper-containing glycoprotein, is the rate-limiting enzyme of the melanogenesis pathway and is commonly used as a therapeutic target to alleviate hyperpigmentation of the skin. However, only a few tyrosinase inhibitors have entered clinical use, owing to safety concerns or weaknesses. Recently, the combination of antioxidants and antityrosinase has been suggested as an effective means of preventing hyperpigmentation [15,16].

*Morus alba* L., known as white mulberry, is widely cultivated in China and other countries. The root bark of *Morus alba* L. (Mori Cortex) is a traditional Chinese medicinal (TCM) herb used to treat diuresis and diabetes [17]. Several studies have demonstrated that *Morus alba* L. possesses various biological activities, including prevention of osteoarthritis, promotion of hair growth, and improvement of immune response, as well as antihyperlipidemic, antibacterial, anticancer, anti-inflammatory, antidiabetic, and antioxidant activities [17,18,19,20,21]. Based on the results of previous studies, we investigated whether mulberry bark affected antioxidant, anti-inflammatory, anti-α-glucosidase, and antityrosinase effects under the influence of various solvent extracts and whether it has potential for further research. In this study, we evaluated the antioxidant, anti-inflammatory, anti-α-glucosidase, and antityrosinase effects of various solvent extracts of root bark of *M. alba* and their bioactive components (morin (**1**), morusin (**2**), oxyresveratrol (**3**), umbelliferone (**4**), kuwanon G (**5**), and kuwanon H (**6**)) for the first time. In addition, the amount of isolated compounds was quantified by high-performance liquid chromatography (HPLC) analysis, and biological activity models were established by molecular docking.

## 2. Materials and Methods

### 2.1. Chemicals and Reagent

Quercetin was purchased from MedChemExpress (Monmouth Junction, NJ, USA). 2,2’-Azino-bis (3-ethylbenzothiazoline-6-sulfonic acid) (ABTS), Folin–Ciocalteu reagent, tyrosinase, gallic acid, *α*-glucosidase, 2,4,6-Tris(2-pyridyl)-s-triazine (TPTZ), bovine serum albumin, and Trolox were purchased from Sigma-Aldrich (St. Louis, MO, USA). Disodium hydrogen phosphate, potassium peroxodisulfate, sodium carbonate, and sodium dihydrogen phosphate were purchased from SHOWA Chemical Co. Ltd. (Chuo-ku, Japan). Ferric chloride (FeCl_3_), *p*-nitro-phenyl-α-D-glucopyranoside (p-NPG), and aluminum chloride (AlCl_3_) were obtained from Alfa Aesar (Lancashire, UK). 2,2-diphenyl-1-(2,4,6-trinitrophenyl) hydrazyl (DPPH), nitroblue tetrazolium (NBT), and phenazine methosulphate (PMS) were supplied by Tokyo Chemical Industry Co., Ltd. (Tokyo, Japan). Potassium acetate, acarbose, sodium acetate, nicotinamide adenine dinucleotide (NADH), and butyl hydroxytoluene (BHT) were acquired from Acros Organics (Geel, Belgium). Arbutin was purchased from AK Scientific. Acetic acid was supplied by Avantor Performance Materials, LLC. (Radnor, PA, USA).

### 2.2. Preparation of Morus alba *L.* Extract

Root barks of *Morus alba* L. were purchased from Neihu Dist., Taipei City, Taiwan, in August 2021 and identified by Prof. J.-J. Chen. A voucher specimen was deposited in the Department of Pharmacy, National Yang Ming Chiao Tung University, Taipei, Taiwan. The root bark of *Morus alba* L. (15 g) was left to soak in 150 mL of various solvents (water, MeOH, EtOH, acetone, EtOAc, dichloromethane, and *n*-hexane) and shaken for 24 h at room temperature with an orbital shaker (model OS701, TKS Company, Taipei, Taiwan). The extracts were filtered through filter paper (Whatman No. 1) and concentrated using a rotary evaporator (EYELA N-N Series, Tokyo Rikakikai Co., Ltd., Tokyo, Japan) connected to a vacuum pump (EYELA NVP-1000, Tokyo Rikakikai Co., Ltd., Tokyo, Japan) at 38 °C. The extracts were stored at –20 °C until further experiments.

### 2.3. Preparation of Active Components

The root barks (1.0 Kg) of *Morus alba* L. were extracted 3 times for 3 days with MeOH (5.0 L). The MeOH extract was concentrated under reduced pressure at 37 °C to obtain MeOH extract (51.6 g). The MeOH extract (fraction A, 51.6 g) was purified by column chromatography (CC) (2.0 kg of reversed-phase C18 silica gel, 200–400 mesh; H_2_O/MeOH 90:1–0:1, 2000 mL) to afford 10 fractions: A1–A10. Fraction A2 (7.2 g) was subjected to CC (220 g of C18 silica gel, 230–400 mesh (40–63 μm); H_2_O/MeOH 19:1–1:1, 600 mL fractions) to afford 10 subfractions: A2-1–A2-10. Part (264.0 mg) of fraction A2-6 was purified by HPLC (Agilent Technology, Waldbronn, Germany) (ODS column, 0.5% acetic acid in H_2_O/acetonitrile 4:1, 2.0 mL min^−1^) to obtain morin (**1**) (32.6 mg) (*t*_R_ 7.8 min). Fraction A2-10 (850 mg) was separated by column chromatography on a Sephadex LH-20 (Merck KGaA, Darmstadt, Germany) and eluted with 100% MeOH to yield 8 fractions (A2-10-1–A2-10-8). Fraction A2-10-5 (320 mg) was purified by HPLC (Agilent Technology, Waldbronn, Germany) (ODS column, 0.1% formic acid in H_2_O/acetonitrile (4:1–1:4), 1.0 mL min^−1^) to afford kuwanon G (**5**) (44.6 mg) (*t*_R_ 56.3 min) and kuwanon H (**6**) (46.2 mg) (*t*_R_ 61.5 min).

Fraction A3 (4.8 g) was chromatographed on C18 silica gel (230–400 mesh, 200 g) and eluted with H_2_O/MeOH (9:1–1:4) to afford 6 fractions (each 800 mL, A3-1–A3-6). Part (362 mg) of fraction A3-5 was purified by HPLC (Agilent Technology, Waldbronn, Germany) (ODS column, 0.1% formic acid in H_2_O/acetonitrile (20:1–0:1), 1.0 mL min^−1^) to afford oxyresveratrol (**3**) (34.5 mg) (*t*_R_ 11.7 min) and morusin (**2**) (22.3 mg) (*t*_R_ 27.7 min). Fraction A7 (5.7 g) was separated by column chromatography on a Sephadex LH-20 (Merck KGaA, Darmstadt, Germany) and eluted with 100% MeOH to yield 9 fractions (A7-1–A7-9). Part (360 mg) of fraction A7-3 was purified by HPLC (Agilent Technology, Waldbronn, Germany) (ODS column, 0.1% formic acid in H_2_O/acetonitrile, 7:3, 2.0 mL min^−1^) to afford umbelliferone (**4**) (31.4 mg) (*t*_R_ 8.9 min). The structures of morin (**1**), morusin (**2**), oxyresveratrol (**3**), umbelliferone (**4**), kuwanon G (**5**), and kuwanon H (**6**) were identified by nuclear magnetic resonance (NMR) spectra acquired using a Bruker Avance 600 MHz spectrometer (Bruker, Bremen, Germany) (Figures S1–S6).

### 2.4. Reverse-Phase HPLC

Reverse-phase HPLC separations were conducted using a mobile phase of acetonitrile (solvent A) and 0.2% acetic acid in water (v/v) (solvent B) as follows: 0–30 min, linear gradient from 2 to 55% A; 30–40 min, linear gradient from 55 to 60% A; 40–70 min, linear gradient from 60 to 100% A; 70–80 min, back to initial 98% B.

### 2.5. Determination of Total Phenolic Content

The total phenolic content (TPC) of the various solvent extracts was determined by the Folin–Ciocalteu method as previously reported [22]. Briefly, 50 μL of each extract or gallic acid was added to a 96-well microtiter plate, mixed with 50 μL of 0.5 N Folin–Ciocalteu reagent, and incubated for 5 min. Subsequently, 100 μL of a 20% Na_2_CO_3_ solution was added and incubated for 40 min at room temperature in the dark. Finally, the absorbance was measured at 750 nm. The TPC of the extracts was determined using a standard calibration curve of gallic acid with an R^2^ value of 0.9995.

### 2.6. Determination of Total Flavonoid Content

The total flavonoid content (TFC) of the various solvent extracts was determined using the aluminum chloride colorimetry method as previously reported [23]. Briefly, 400 μL of the extract sample or quercetin was mixed with 200 μL of 10% (*w*/*v*) aluminum chloride solution and 200 μL of 0.1 mM potassium acetate solution and reacted for 30 min at room temperature. Then the absorbance was measured at 415 nm. The TFC of the extract was determined by a standard calibration curve of quercetin with an R^2^ value of 0.9991.

### 2.7. DPPH Radical Scavenging Activity

This assay was performed using a previously reported procedure [24]. This experiment needs to be performed in the dark. Briefly, 100 µL of varying concentrations of extracts or compounds were mixed with 100 µL of 200 µM DPPH solution and incubated at room temperature for 30 min, and the absorbance was measured at 520 nm. DPPH radical scavenging activity was calculated using the following equation.

DPPH scavenging activity (%) = (A_0_ − A_1_)/A_0_ × 100, where A_1_ is the absorbance of the test sample, and A_0_ is the absorbance of the control (untreated group).

### 2.8. ABTS Radical Scavenging Activity

This assay was performed using a previously reported procedure [25]. This experiment needs to be performed in the dark. First, the reaction reagent was prepared by mixing 28 mM ABTS solution and 9.6 mM potassium persulfate solution (v/v, 1/1) and left in the dark at 4 °C for 16 h before use. The reaction reagent was diluted with ethanol until the absorbance was 0.70 ± 0.02 at 740 nm. Next, 10 μL of the extract or compound was mixed with 190 μL of the reaction reagent and incubated at room temperature for 5 min before measuring the absorbance at 740 nm. The formula of scavenging activity was calculated as follows.

ABTS radical scavenging activity (%) = (A_0_ − A_1_)/A_0_ × 100, where A_0_ and A_1_ are the absorbance of the control (untreated group) and test sample, respectively.

### 2.9. Superoxide Radical Scavenging Activity

This assay was performed using a previously reported procedure [24]. This experiment needs to be performed in the dark. Initially, NBT (300 μM), NADH (468 μM), and PMS (120 μM) were prepared using Tris-HCl buffer (16 mM, pH 8.0). Next, 50 μL of NBT and PMS and 10 μL of the extract or compound were mixed; subsequently, 50 μL of NADH solution was added and incubated at room temperature for 5 min before measuring the absorbance at 560 nm. The equation for the scavenging activity is as follows.

Superoxide radical scavenging activity = (A_0_ − A_1_)/A_0_ × 100, where A_0_ and A_1_ are the absorbance of the control (untreated group) and test sample, respectively.

### 2.10. Ferric Reducing Antioxidant Power (FRAP)

The FRAP assay was performed using a previously described procedure [26]. This experiment needs to be performed in the dark. First, the reaction reagent was obtained by mixing acetate buffer (pH 3.6), ferric chloride solution (20 mM), and TPTZ solution (10 mM TPTZ in 40 mM HCl) in a ratio of 10:1:1, respectively. Next, 900 μL of the reaction reagent was mixed with 100 μL of the sample or Trolox in an Eppendorf tube, and the absorbance was measured at 593 nm after 40 min in a dry bath at 37 °C. The FRAP of the extracts was determined by a linear standard calibration curve from 0 to 100 mM Trolox with an R^2^ value of 0.9999.

### 2.11. α-Glucosidase Inhibitory Activity Assay

The α-glucosidase inhibitory assay was conducted on the basis a previously described method [27]. Briefly, 100 μL of extract or compound was mixed with 20 μL of 1 U/mL *α*-glucosidase solution in an Eppendorf tube. Subsequently, 380 μL of 0.53 mM p-NPG was added and incubated in a dry bath at 37 °C for 30 min. Finally, 500 μL of 0.1 M Na_2_CO_3_ solution was added to terminate the reaction, and the absorbance was measured at 405 nm. The inhibition was calculated as follows.

α-Glucosidase inhibition (%) = (A_0_ − A_1_)/A_0_ × 100, where A_0_ and A_1_ are the absorbance of the control (untreated group) and test sample, respectively.

### 2.12. Tyrosinase Inhibitory Activity Assay

The tyrosinase inhibitory assay was conducted on the basis of a previously described method [28]. The reagents for this experiment were all configured with potassium phosphate buffer (50 mM, pH 6.5). In brief, 80 μL of L-tyrosine was mixed with 100 μL of the extract or compound at room temperature and incubated for 10 min. After incubating with 20 μL of 1000 U/mL tyrosinase for 25 min, the absorbance was measured at 490 nm. The inhibition was calculated as follows.

Tyrosinase inhibition (%) = (A_0_ − A_1_)/A_0_ × 100, where A_0_ and A_1_ are the absorbance of the control (untreated group) and test sample, respectively.

### 2.13. Cell Culture

Murine RAW264.7 macrophages were cultured at 37 °C under 5% CO_2_ using a culture medium that was a mixture of DMEM, 10% FBS, and 1% penicillin [29]. When the cells reached seven or eight percent full growth, the seeded cells were subjected to NO measurement and iNOS protein assay.

### 2.14. Nitric Oxide Inhibitory Assay

The NO inhibition assay was performed as described in [29], with slight modification. RAW264.7 cells were seeded in 96 wells for 24 h. The next day, they were divided into the following groups: medium, medium with LPS (100 ng/mL) added, medium with the positive control (quercetin) and LPS added, and medium with samples and LPS (100 ng/mL) added. The reaction time between the medium and the sample was 1 h before adding LPS and waiting 20 h after adding LPS. Finally, half of the supernatant was transferred into a new 96-well plate and mixed in a 1:1 ratio with Griess reagent. After 15 min in the dark, the reaction was measured at 550 nm.

### 2.15. MTT Assay

The MTT assay was performed according to the reference method, with slight modifications [29]. Cells from the 96-well plates prepared for the NO inhibition assay were added to MTT stock solution (5 mg/mL) and diluted 20-fold with medium. After 2 h, all liquid was removed, and DMSO was added. The absorbance was measured at 570 nm.

### 2.16. Western Blot Analysis

Western blot analysis was performed according to the reference method, with slight modifications [29]. Cells were cotreated with varying concentrations of samples and LPS (100 ng/mL) for 24 h. After washing the cells with ice-cold PBS, the proteins were collected with cell lysis buffer and stored at −20 °C for subsequent experiments. The quantified proteins were separated using a 10% SDS-polyacrylamide gel. Next, the proteins were transferred to polyvinylidene fluoride (PVDF) membranes by electrophoresis. The transferred PVDF membranes were immersed in 2% BSA blocking buffer for 2 h and washed two to three times with TBST. Proteins were visualized by specific primary antibodies and soaked overnight. The next day, PVDF membranes were subjected to the TBST wash step again to remove residual primary antibodies and soaked with secondary antibodies for 2 h. Finally, after repeating the TBST wash two to three times, immunoreactivity was detected with ECL reagents, and samples were exposed with a cold light meter and quantified with Image J.

### 2.17. Molecular Modeling Docking Study

The crystal structures of α-glucosidase (PDB: 3A4A), tyrosinase (PDB: 2Y9X), and inducible nitric oxide synthase (PDB: 1M9T) were retrieved from the Protein Data Bank and prepared for the docked receptors by adding hydrogen atoms and Gasteiger charge measurements via AutodockTools (ADT ver. 1.5.6) (Scripps Research, San Diego, CA, USA). The 3D structures of the ligands were constructed in the Chem3D program Ultra 12.0 (PerkinElmer, Cambridge, MA, USA). Hydrogen addition, Gasteiger charge measurements, and selection of flexible torsions for ligands were optimized by AutodockTools (ADT ver. 1.5.6) (Scripps Research, San Diego, CA, USA). Molecular docking studies were performed using AutoDock Vina software (ADT ver. 4.0.1) (Scripps Research, San Diego, CA, USA) [30]. The grid dimensions of α-glucosidase (PDB: 3A4A) were designed as 30 Å × 30 Å × 30 Å for kuwanon H (**6**) and 18 Å × 18 Å × 18 Å for acarbose. The grid dimensions of tyrosinase (PDB: 2Y9X) were designed as 30 Å × 30 Å × 30 Å for oxyresveratrol (**3**) and 30 Å × 30 Å × 30 Å for arbutin. The grid dimensions of inducible nitric oxide synthase (PDB: 1M9T) were designed as 20 Å × 18 Å × 18 Å for morusin (**2**) and 16 Å × 16 Å × 16 Å for quercetin. Binding affinity energies are displayed in kcal/mol for their docking scores. Visualization of the optimal docking interactions was analyzed using Biovia Discovery Studio Client 2021 (DS Biovia, San Diego, CA, USA) [31].

### 2.18. Statistical Analysis

All data are expressed as mean ± SEM. Statistical analysis was conducted using Student’s *t*-test. A probability of 0.05 or less was considered statistically significant. All experiments were performed at least 3 times.

## 3. Results

### 3.1. Determination of TPC, TFC and Yields in Each Solvent Extract

We studied solvent extracts from the root bark of *Morus alba* L. for their TPC, TFC, and yields. Table 1 shows the TPCs, TFCs, and yields assessed for water, methanol, ethanol, acetone, ethyl acetate, dichloromethane, and *n*-hexane extracts. The various solvent extracts yielded from 1.5 ± 0.49% (*n*-hexane) to 23.6 ± 5.50% (water extract). The yields of the water extract were significantly higher those of the other extracts. Moreover, the results showed that the yield of *Morus alba* L. gradually decreased from high polarity to low polarity. Among the tested solvent extracts, the results showed that the highest TPC was contained in acetone extract (94.61 ± 2.36 mg/g), followed by EtOAc (68.65 ± 6.12 mg/g) and MeOH extracts (60.76 ± 1.41 mg/g). Apart from this, the TFC were similar to the TPC results of acetone and EtOAc extracts. The acetone extract (167.22 ± 1.26 mg/g) still had the highest TFC among all tested extracts, followed by ethyl acetate (156.20 ± 1.75 mg/g) and ethanol extracts (117.77 ± 2.01 mg/g).

### 3.2. DPPH Free Radical Scavenging Activity

Table 2 shows the DPPH radical scavenging activity of the various extracts of *Morus alba* L. Although the results show that none of the extracts were not superior to the positive control (BHT, SC_50_ = 19.75 ± 1.79 μg/mL), among the various solvent extracts, acetone extract (SC_50_ = 242.33 ± 15.78 μg/mL) displayed the highest DPPH radical scavenging activity, followed by MeOH (SC_50_ = 308.10 ± 8.24 μg/mL) and EtOH extracts (SC_50_ = 331.81 ± 22.55 μg/mL).

### 3.3. ABTS Free Radical Scavenging Activity

As shown in Table 2, although the free radical scavenging activity of the various solvent extracts against ABTS was lower than that of the positive control (BHT, SC_50_ = 25.87 ± 5.83 μg/mL), values were higher than that of DPPH. MeOH extract exhibited relatively effective ABTS free radical scavenging activity (SC_50_ = 65.60 ± 6.03 μg/mL), followed by EtOH (SC_50_ = 118.25 ± 3.29), acetone (SC_50_ = 129.28 ± 10.53 μg/mL), EtOAc (SC_50_ = 159.16 ± 10.74 μg/mL), and water extracts (SC_50_ = 257.38 ± 32.53 μg/mL).

### 3.4. Superoxide Radical Scavenging Activity

The results are shown in Table 2. Only the acetone extract exhibited a significant effect in the superoxide radical scavenging test (SC_50_ = 124.80 ± 22.09 μg/mL), followed by MeOH (SC_50_ = 299.13 ± 26.45 μg/mL) and EtOAc extracts (SC_50_ = 388.74 ± 10.26 μg/mL). Other solvent extracts had no significant effect (SC_50_ > 400 μg/mL).

### 3.5. Ferric Reducing Antioxidant Power

As shown in Table 2, the acetone extract displayed the highest ferric reducing antioxidant capacity (TE = 213.67 ± 2.06 mM/g), followed by MeOH extract (TE = 200.51 ± 2.53 mM/g), EtOAc (TE = 197.31 ± 2.11 mM/g), and EtOH extracts (TE = 181.83 ± 3.24 mM/g). Additionally, dichloromethane (TE = 98.39 ± 0.69 mM/g), water (TE = 52.32 ± 1.91 mM/g), and *n*-hexane extracts (TE = 50.83 ± 0.91 mM/g) showed relatively low reducing capacities.

Based on the results of the above antioxidant assay, the acetone extract of *Morus alba* L. exhibited strongest antioxidant capacity among all solvent extracts, including DPPH, superoxide radical scavenging activity, and FRAP test. This result may be attributed to the acetone extract having the highest phenolic content. In addition to the acetone extract, MeOH extract also displayed significant effects in the antioxidant tests (ABTS radical scavenging activity and FRAP test).

### 3.6. Anti-α-Glucosidase Activity Assay

The results listed in Table 3 show that the acetone extract of *Morus alba* L. demonstrated the greatest anti-α-glucosidase activity (IC_50_ = 3.87 ± 1.95 μg/mL), followed by EtOAc (IC_50_ = 5.80 ± 2.29 μg/mL), EtOH (IC_50_ = 11. 48 ± 1.81 μg/mL), dichloromethane (IC_50_ = 12.04 ± 6.67 μg/mL), MeOH (IC_50_ = 12.48 ± 0.93 μg/mL), water (IC_50_ = 17.94 ± 6.37 μg/mL), and *n*-hexane extracts (IC_50_ = 22.85 ± 4.51 μg/mL). All these solvent extracts showed higher inhibitory activity against *α*-glucosidase than the antidiabetic drug acarbose (IC_50_ = 355.48 ± 28.39 μg/mL).

### 3.7. Antityrosinase Activity Assay

Among all the extracts, as shown in Table 3, the acetone extract of *Morus alba* L. (IC_50_ = 7.95 ± 1.54 μg/mL) showed the strongest antityrosinase activity, followed by ethyl acetate (IC_50_ = 11.27 ± 2.75 μg/mL), methanol (IC_50_ = 13.41 ± 0.77 μg/mL), and ethanol extracts (IC_50_ = 19.86 ± 3.35 μg/mL). The other three solvent extracts exhibited no significant inhibitory activity (IC_50_ > 400 μg/mL).

In our study, the anti-α-glucosidase and antityrosinase activities of various solvent extracts of *Morus alba* L. (water, methanol, ethanol, acetone, ethyl acetate, dichloromethane, and *n*-hexane) were comparatively assessed for the first time. Based on the results of the above two tests, acetone and ethyl acetate of *Morus alba* L. were the extracts with the most potential *α*-glucosidase and tyrosinase inhibitory activity.

### 3.8. Anti-Inflammatory Activity of Solvents

**Figure 1:**
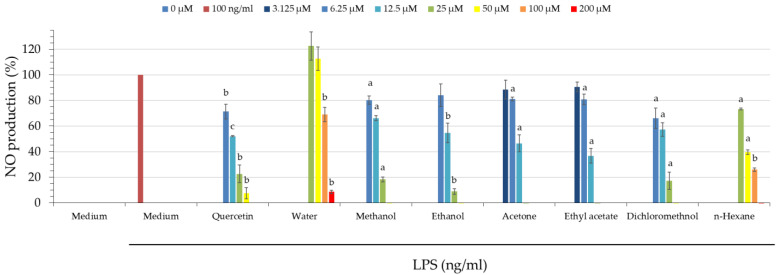

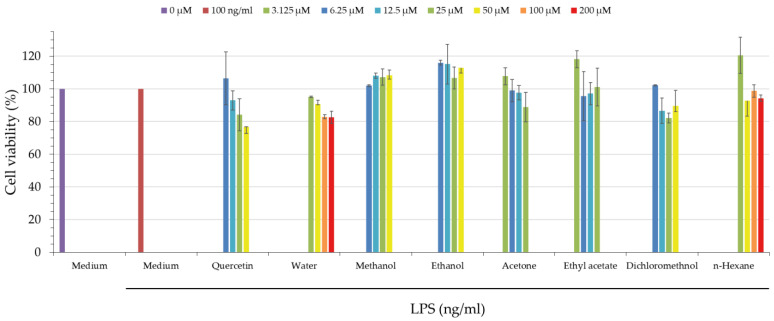
The effect of various extracts from *Morus alba* L. on cell viability of RAW 264.7 cells was assessed by MTT assay. Values are expressed as mean ± SD (*n* = 3); quercetin was used as a positive control. ^a^ *p* < 0.05, ^b^ *p* < 0.01, and ^c^ *p* < 0.001 compared to the LPS-induced group.

Nitric oxide (NO) is considered a proinflammatory mediator that induces inflammation, and selective NO biosynthesis inhibitors are indicated to treat NO-induced inflammation [32,33]. Therefore, NO inhibitors are regarded as an important therapeutic advance in inflammatory diseases. As shown in Table 4 and Figure 1, among all extracts, ethyl acetate (IC_50_ = 10.81 ± 1.41 μg/mL) and acetone extracts (IC_50_ = 12.00 ± 1.32 μg/mL) of *Morus alba* L. showed the most potent anti-inflammatory activity, followed by ethanol (IC_50_ = 14.30 ± 2.38 μg/mL), dichloromethane (IC_50_ = 15.33 ± 2.63 μg/mL), methanol (IC_50_ = 17.19 ± 2.04 μg/mL), *n*-hexane (IC_50_ = 53.22 ± 17.28 μg/mL), and water extracts (IC_50_ = 121.08 ± 7.85 μg/mL).

### 3.9. Cell Viability of Extracts

The effect of different solvent extracts on the cell viability of RAW 264.7 cells was assessed by MTT assay to determine their cytotoxicity. As shown in Figure 1, there was no significant difference between the LPS-induced group and the control group (non-LPS-induced). The results of the different solvent extracts from *Morus alba* L. showed cell viability above 80%, which means that they were not cytotoxic. In contrast, quercetin showed a slight cytotoxicity at higher doses.

### 3.10. Quantitation of Active Components in Solvent Extracts

**Figure 2:**
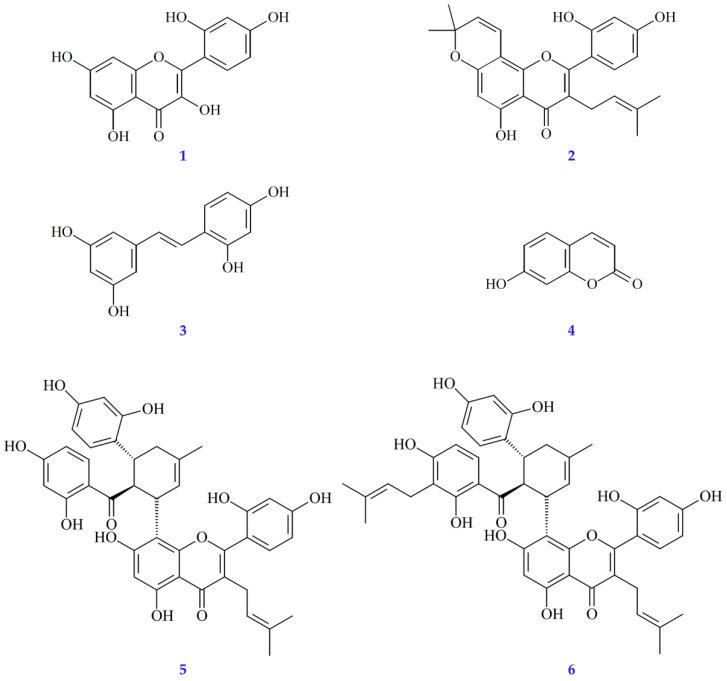
Chemical structures of morin (**1**), morusin (**2**), oxyresveratrol (**3**), umbelliferone (**4**), kuwanon G (**5**), and kuwanon H (**6**) from *Morus alba*.

The active compounds in the investigated solvent extracts of *Morus alba* L. were quantified by reverse-phase HPLC analysis (Figures S5–S12). The amounts of the six active compounds (morin (**1**), morusin (**2**), oxyresveratrol (**3**), umbelliferone (**4**), kuwanon G (**5**), and kuwanon H (**6**)) (Figure 2) in the solvent extracts are shown in Table 5. The total amounts of the six active compounds in the solvent extracts ranged from 22.87 ± 1.77 mg/g (*n*-hexane extract) to 63.62 ± 5.71 mg/g (methanol extract) in the order of methanol > ethanol > acetone > ethyl acetate > water > dichloromethane > *n*-hexane extract. Furthermore, in seven solvent extracts, kuwanon G (5) was the most abundant of the six active compounds, followed by kuwanon H (**6**), morusin (**2**), morin (**1**), oxyresveratrol (**3**), and umbelliferone (**4**).

### 3.11. Antioxidant Activities of Isolated Components

The isolated compounds, morin (**1**), morusin (**2**), oxyresveratrol (**3**), umbelliferone (**4**), kuwanon G (**5**), and kuwanon H (**6**) (Figure 1), were measured for their antioxidant effects, including ABTS, DPPH, superoxide radical scavenging activities, and FRAP assay. Results are shown in Table 6. Compound **1** exhibited the strongest antioxidant effects among all tested compounds. Compounds **1** (SC_50_ = 17.49 ± 3.43 μM) and **3** (SC_50_ = 44.42 ± 12.51 μM) showed higher radical scavenging activities than the positive control, BHT (SC_50_ = 89.84 ± 7.03 μM) in DPPH. In ABTS, compound **6** (SC_50_ = 8.68 ± 0.93 μM) exhibited the strongest radical scavenging activity, followed by **5** (SC_50_ = 9.28 ± 1.02 μM), **3** (SC_50_ = 9.50 ± 2.08 μM), **1** (SC_50_ = 12.67 ± 5.98 μM), and **2** (SC_50_ = 44.19 ± 8.07 μM). All of these compounds exhibited higher radical scavenging activity than BHT (SC_50_ = 115.86 ± 25.14 μM). Compound **1** (SC_50_ = 54.50 ± 18.96 μM) exhibited more potent antioxidant activity than other isolated compounds in superoxide radical scavenging assays. The antioxidant activity of isolated compounds was also evaluated by FRAP assay and compared with BHT (TE = 4385.56 ± 78.88 mM/g); compounds **5** (TE = 3549.22 ± 160.65 mM/g), **1** (TE = 2963.28 ± 63.65 mM/g), and **3** (TE = 2402.22 ± 32.91 mM/g) showed moderate antioxidant power.

### 3.12. Anti-α-Glucosidase Activities of Isolated Components

The *α*-glucosidase inhibitory activity of six isolated compounds from *Morus alba* L. is shown in Table 7. The results show that the inhibitory activity of all isolated compounds was stronger than that of the positive control, acarbose (IC_50_ = 540.38 ± 47.42 μM). Kuwanon H (**6**) (IC_50_ = 4.06 ± 0.12 μM) exhibited significant inhibitory activity against α-glucosidase (2–20 times more potent than other compounds), followed by compounds **5** (IC_50_ = 7.00 ± 0.31 μM), **3** (IC_50_ = 8.35 ± 0.46 μM), **2** (IC_50_ = 15.66 ± 2.15 μM), and **1** (IC_50_ = 90.15 ± 10.17 μM).

### 3.13. Antityrosinase Activities of Isolated Components

The tyrosinase inhibitory activity of six isolated compounds from *Morus alba* L. is shown in Table 7. The results show that in terms of the inhibitory activity of the isolated compounds, that of oxyresveratrol (**3**) (IC_50_ = 1.89 ± 0.59 μM) was 100 times stronger than the positive control, arbutin (IC_50_ = 206.08 ± 36.50 μM). Furthermore, compound **5** (IC_50_ = 100.40 ± 9.62 μM) showed moderate antityrosinase activity, and compounds **1** (IC_50_ = 199.41 ± 84.44 μM) and **6** (IC_50_ = 218.00 ± 40.96 μM) showed a similar tyrosinase inhibitory activity as arbutin.

### 3.14. Anti-Inflammatory Activities of Isolated Components

**Figure 3:**
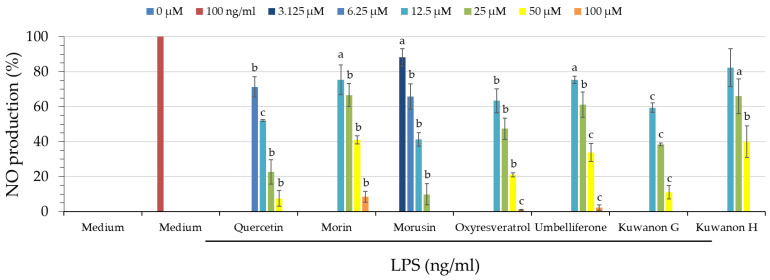

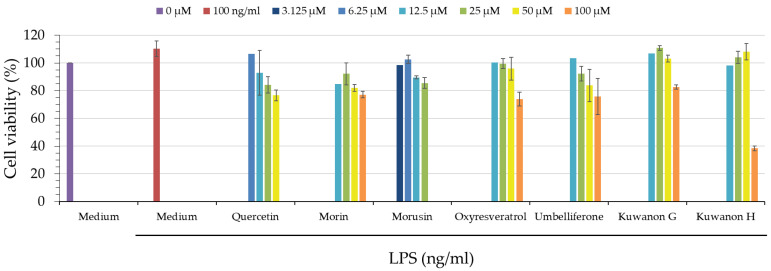
The effect of isolated compounds from *Morus alba* L. on cell viability of RAW 264.7 cells was assessed by MTT assay. Values expressed as mean ± SD (*n* = 3); quercetin was used as a positive control. ^a^ *p* < 0.05, ^b^ *p* < 0.01, and ^c^ *p* < 0.001 compared to the LPS-induced group.

The nitric oxide (NO) production inhibitory activity of six isolated compounds from *Morus alba* L. is shown in Table 8 and Figure 3. Morusin (**2**) (IC_50_ = 9.87 ± 0.59 μM) displayed stronger anti-NO activity than the positive control, quercetin (IC_50_ = 13.20 ± 2.00 μM). In addition, kuwanon G (**5**) (IC_50_ = 17.80 ± 0.50 μM), oxyresveratrol (**3**) (IC_50_ = 25.36 ± 3.47 μM), umbelliferone (**4**) (IC_50_ = 36.76 ± 2.26 μM), kuwanon H (**6**) (IC_50_ = 40.48 ± 4.38 μM), and morin (**1**) (IC_50_ = 43.26 ± 4.61 μM) showed moderate anti-NO activities.

### 3.15. Cell Viability of Isolated Components

The isolated compounds were assessed for their cytotoxic effects on RAW 264.7 cells by MTT assay. As shown in Figure 3, there was no significant difference between the LPS-induced group and the control group (non-LPS-induced). Quercetin was used as a positive control and showed a slight cytotoxicity at higher doses. The results for the isolated compounds showed cell viability above 80%, except for the high dose of 100 μM, which means they were not cytotoxic.

### 3.16. Western Blot Analysis of Isolated Components

**Figure 4:**
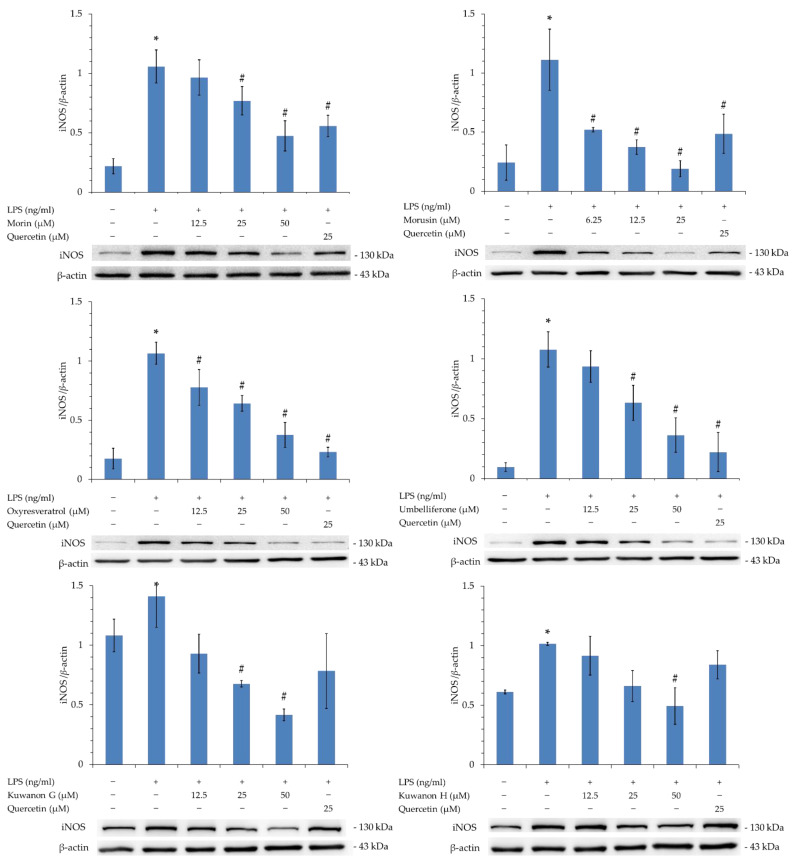
The isolated components were analyzed for iNOS expression by Western blotting. iNOS/β-actin quantification data are expressed as mean ± SEM; quercetin was used as a positive control; * *p* < 0.05 compared to the control group, ^#^ *p* < 0.05 compared to the LPS group.

According to the NO production inhibitory activity (Table 8), the isolated compounds were further evaluated by Western blot for their inhibitory activity on inducible nitric oxide synthase (iNOS). As shown in Figure 4, there was a significant difference between the LPS-induced group and the control group. Morusin (**2**) was the most effective compound, significantly reducing the expression of iNOS compared to the LPS-induced group and showing better increased activity compared to quercetin at 25 μM. The other three compounds significantly reduced the expression of iNOS compared to the LPS-induced group and showed similar inhibitory activity to quercetin (25 μM) at a high dose of 50 μM. The results show that the effects of the isolated compounds were dose-dependent, with significant inhibitory activity against iNOS.

### 3.17. Molecular Modeling Docking

Based on the results of the anti-*α*-glucosidase assay (Table 7), kuwanon H (**6**) showed the strongest inhibitory activity against *α*-glucosidase among all isolates. Therefore, compound **6** was used for molecular docking models, which were generated using the Discovery Studio 2021 (Accelrys, San Diego, CA, USA) modeling program to understand its ability to bind to *α*-glucosidase. Owing to the lack of 3D crystal structure of *Saccharomyces cerevisiae α*-glucosidase, the crystal structure from *Saccharomyces cerevisiae* (PDB: 3A4A) has 72% sequence homology to *Saccharomyces cerevisiae α*-glucosidase and is commonly used for docking studies. In the present study, the 3D crystal structure (PDB: 3A4A) was used as a docking model, as its active site is similar in conformation to α-glucosidase from beta vulgaris, although it is deep and narrow [34].

**Figure 5:**
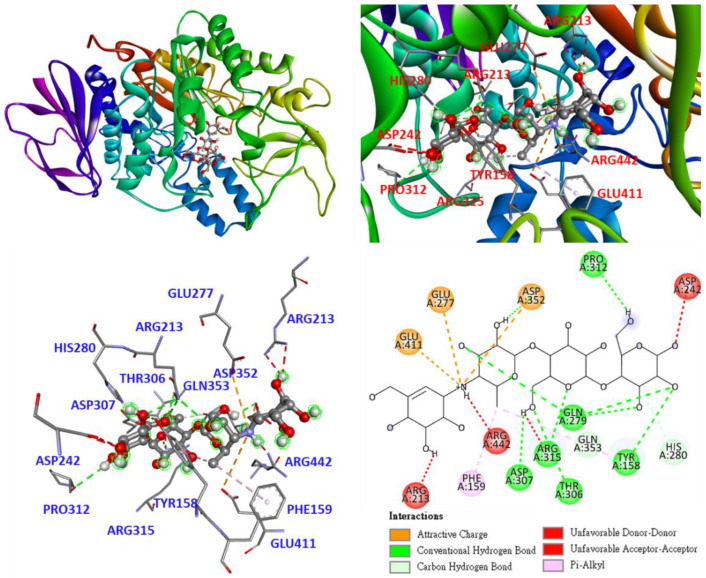
Interaction of acarbose with active sites of *S. cerevisiae α*-glucosidase.

First, as shown in Figure 5, acarbose enters the deep pocket of the active site and lies horizontally in the pocket. Furthermore, the interaction between acarbose and *α*-glucosidase shows that the hydroxyl group of acarbose A-ring deep in the pocket serves as an H-bond donor interacting with ARG213 as an unfavorable donor. The amines that link the A and B rings interact with ARG442 as unfavorable donor donors, as well as with GLU277, GLU411, and ASP352 as attractive charges. Hydroxyl groups on rings B, C, and D serve as H-bond donors and H-bond acceptors and interact with TYR158, GLN279, THR306, ASP307, PRO312, ARG315, and ASP352 as conventional hydrogen bonds, as well as with HIS280 and GLN353 as carbon hydrogen bonds.

The structures of polysaccharides and acarbose show obvious similarities, but one notable difference is the linkage between the A and B rings. The mechanism of *α*-glucosidase suggests that the conversion of polysaccharides to monosaccharides involves the breaking of the *α*-1, 4-glycosidic bond; however, on acarbose, it is nitrogen rather than oxygen. The H-bond interaction of nitrogen is stronger than that of oxygen; therefore, acarbose is able to stay at the active site for a longer time and has the ability to inhibit alpha-glucosidase.

**Figure 6:**
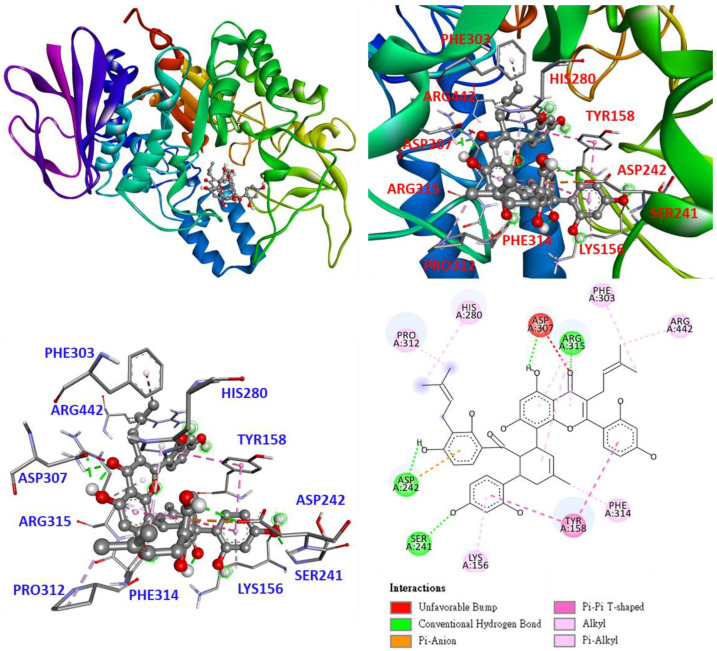
Interaction of kuwanon H (**6**) with active sites of *S. cerevisiae α*-glucosidase.

As shown in Figure 6, kuwanon H (**6**) enters the active site as part of the flavonoid structure and lies deep in the pocket. Whereas the benzene ring interacts with TYR158 in π-π T-shaped interactions. 3-(3-methylbut-2-enyl) forms π-alkyl and alkyl interactions with PHE303 and ARG442, respectively; the chromone structure form conventional hydrogen bonds with ARG315 and ASP307, respectively, and π-alkyl and unfavorable bump interactions with ARG315 and ASP307, respectively. 8-[(1S,5R,6S)-3-methyl-1-cyclohex-2-enyl] forms π-alkyl interactions with PHE314; 8-[(1S,5R,6S)-5-(2,4-dihydroxyphenyl)] forms conventional hydrogen bonds, π-alkyl, and π-π T-shaped interactions with SER241, LYS156, and TYR158, respectively; 8-[(1S,5R,6S)-6-[2,4-dihydroxy-3-(3-methylbut-2-enyl)benzoyl] interacts with ASP242, PRO312, and HIS280 to form conventional hydrogen bonds, as well as π-anion, alkyl, and π-alkyl interactions.

According to the data presented in Table 9, the binding affinity of compound **6** was significantly higher than that of other compounds, indicating that compound **6** has a good binding capacity for *α*-glucosidase, which is consistent with the data presented in Table 7. The binding affinities of the other compounds were also consistent with the data presented in Table 7. The results of this study suggest that active compound **6** may be worthy of further investigation as a natural *α*-glucosidase inhibitor.

Based on the experimental data shown in Table 7, the most potent compound, oxyresveratrol (**3**), was determined by molecular docking for its ability to bind to the crystal structure of tyrosinase from *Agaricus bisporus*. The 3D structure of tyrosinase (PDB: 2Y9X) used as a docking model is from *Agaricus bisporus*, and the substrate binding site consists of five α-helices and several loops, which mainly contain hydrophilic residues interacting with two copper ions, such as six histidines, His 61, 85, 94, 259, 263, and 296 [35].

**Figure 7:**
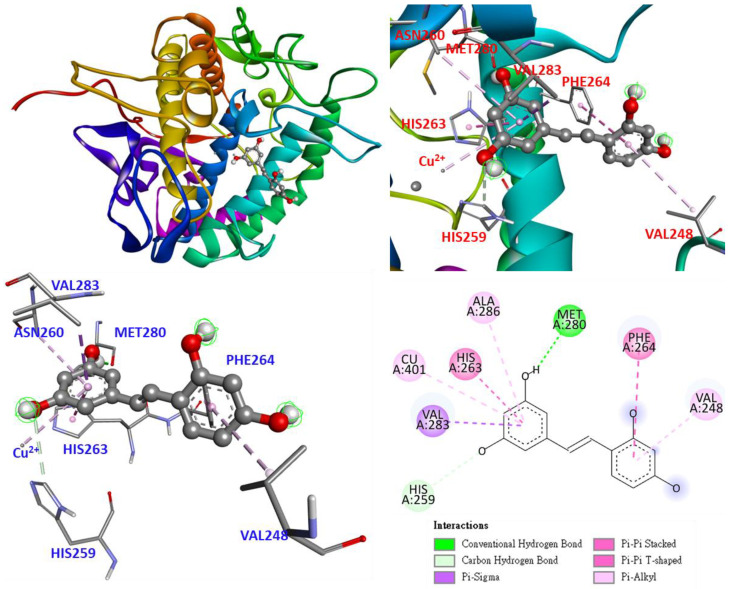
Interaction of oxyresveratrol (**3**) with active sites of *Agaricus bisporus* tyrosinase.

As shown in Figure 7, the docking model of oxyresveratrol (**3**) demonstrates that oxyresveratrol resides in the substrate binding pocket parallel to HIS263 using the benzene ring structure. The benzene ring interacts with the copper ion and ALA286 as π-alkyl. The benzene ring interacts with HIS263 and VAL283 as π-π stacked and π-sigma, respectively. The hydroxyl groups on the benzene ring serve as H-bond donors and H-bond acceptors to form a conventional hydrogen bond with MET280 and a carbon–hydrogen bond with HIS259, respectively. The other benzene ring interacts with PHE264 and VAL248 as π-π T shaped and π-alkyl interactions, respectively.

**Figure 8:**
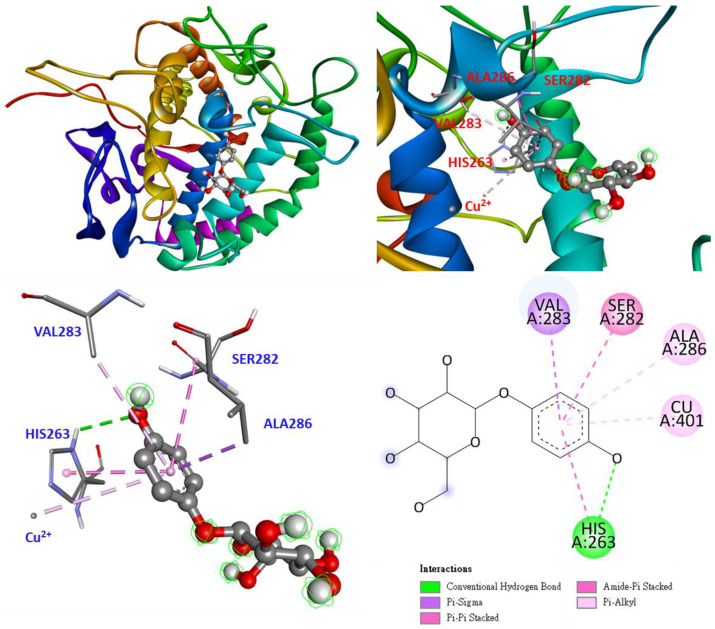
Interaction of arbutin with active sites of *Agaricus bisporus* tyrosinase.

As shown in Figure 8, the positive control, arbutin, enters as a benzene ring in the substrate binding pocket, and the benzene ring and hydroxyl group interact in parallel to HIS263, forming a π-π stacked and conventional hydrogen bond. Besides interacting with the copper ion and ALA286 as π-alkyl, the benzene ring interacts with VAL283 and SER282 as π-sigma and amide-π stacked bonds, respectively. The part of arbutin’s glycosyl ring is probably not as long as compound **3**, owing to its smaller size. Therefore, arbutin does not interact with any amino acids on the loop.

According to the data presented in Table 10, the binding affinity of compound **3** was significantly higher than that of other compounds and arbutin, indicating that compound **3** has a good binding capacity for tyrosinase, which is consistent with the data presented in Table 7. The binding affinities of compounds **1** and **5** are also consistent with the data presented in Table 7. In this study, active compound **3** not only exhibited antityrosinase activity but also exhibited good binding ability to the active site of tyrosinase, suggesting that compound **3** may be worthy of further investigation as a natural tyrosinase inhibitor.

Based on the results of the NO production inhibition assay (Table 8) and the Western blot results of iNOS (Figure 4), the active compound morusin (**2**) has anti-inflammatory potential. This compound was therefore used in a molecular docking model to determine its ability to bind to iNOS. The 3D structure of iNOS (PDB: 1M9T) used as a docking model is from *Mus musculus*, and the active site consists of four pockets, of which the substrate binding S pocket contains heme [36,37].

**Figure 9:**
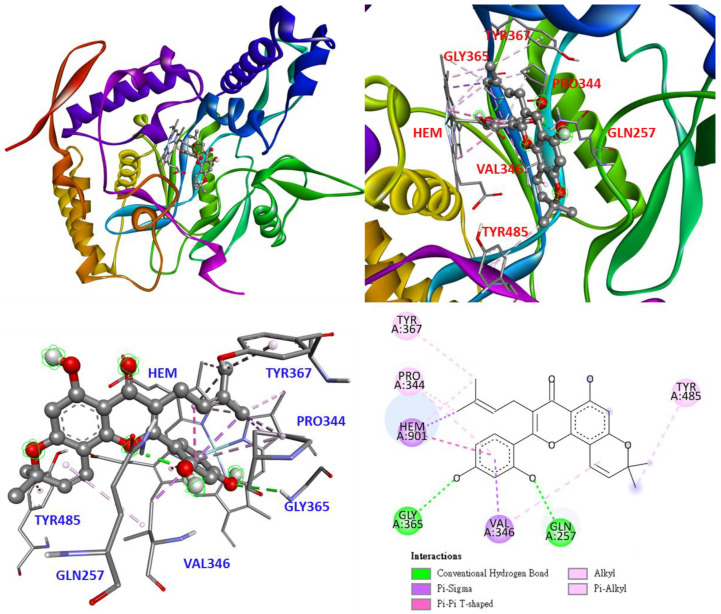
Interaction of morusin (**2**) with active sites of *Mus musculus* iNOS.

As shown in Figure 9, in the molecular docking model for morusin (**2**), morusin uses its 2,4-dihydroxyphenyl and 3-methylbut-2-enyl to stay in the S pocket and interact with heme. 2, 4-Dihydroxyphenyl forms a π-sigma interaction with VAL346, and the hydroxyl group serves as an H-bond acceptor to form a conventional hydrogen bond with GLY365 and GLN257. 3-Methylbut-2-enyl interacts with TYR367 and PRO344 to form π-alkyl and alkyl. In addition, 8-dimethyl forms a π-alkyl interaction with TYR485.

**Figure 10:**
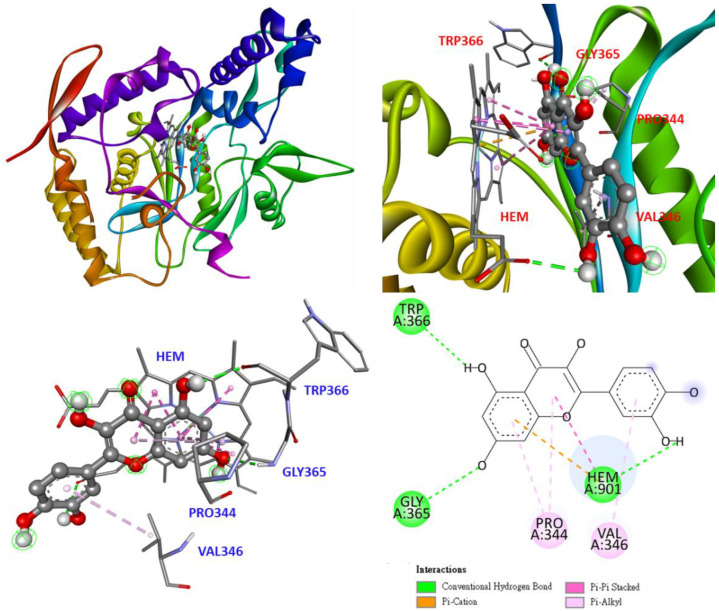
Interaction of quercetin with active sites of *Mus musculus* iNOS.

The docking model of quercetin, the positive control, is shown in Figure 10. The quercetin stays in the S pocket parallel to the heme and forms π-π stacked interactions. The hydroxyl group on quercetin forms a conventional hydrogen bond with TRP366, GLY365, and heme. In addition, the flavonoid backbone of quercetin forms π-alkyl interactions with PRO344 and VAL346.

According to the data presented in Table 11, the binding affinity of compound **2** was significantly higher than that of compounds **1**, **3**, **4**, **5**, and **6**, as well as that of the positive control, quercetin. The results indicate that compound **2** has the ability to bind to iNOS, which is consistent with the anti-NO data presented in Table 8. The binding affinities of the other compounds (in descending order) are **5**, **3**, **6**, **1**, and **4**. All isolated compounds (especially **2**) exhibited potent anti-iNOS activity, suggesting that these compounds deserve further investigation as natural inhibitors of iNOS.

## 4. Discussion

In situations in which many side effects and drug resistance occur, herbal medicine may be a useful alternative treatment. Consequently, natural products (i.e., herbs, plants, and fungi) are gaining international popularity as a source of medicines due to their easy availability and economic viability. Extraction of medicinal plants is the process of deriving active components or secondary metabolites using appropriate solvents [38]. In addition, exogenous factors may affect the extraction of active components, including the nature of the plant material, the solvent used, the pH of the solvent, and the temperature [39]. Therefore, solvents with varying polarities were used to obtain and assess the compound activity of *Morus alba* L. The results show each solvent extract and compound exhibited varying levels of biological activity.

In the present study, DPPH, ABTS, superoxide radical scavenging, and FRAP assays were used to assess the antioxidant potential of various solvent extracts [40,41]. Acetone extract of *Morus alba* L. showed the highest antioxidant activity, a result consistent with the TPC in the extract. Therefore, the difference between the antioxidant activities of different solvent extracts may be due to the varying levels of TPC or antioxidant components. Our study is the first to comparatively evaluate the TPC, TFC, and antioxidant capacity of various solvents (*n*-hexane, dichloromethane, ethyl acetate, acetone, ethanol, methanol, and water) of *Morus alba* L. These results can be used to determine the most suitable solvent for the most efficient extraction of TPC, TFC, and antioxidants. In addition, the isolated compounds were tested for their antioxidant potential. According to the results, compounds **1** and **3** showed strong antioxidant activity compared to the positive control (BHT).

*α*-Glucosidase is located in the epithelial mucosa of the small intestine and has been suggested as a therapeutic target for the regulation of postprandial hyperglycemia. Inhibition of intestinal *α*-glucosidase delays the digestion and absorption of carbohydrates, thereby suppressing postprandial hyperglycemia [42,43]. In the anti-*α*-glucosidase assay, solvent extracts of *Morus alba* L. exhibited stronger *α*-glucosidase inhibitory activity than the positive control, acarbose. In addition, compounds **1**, **2**, **3**, **5**, and **6** showed stronger *α*-glucosidase inhibitory activity than acarbose. Compound 6 was approximately 100 times more effective than acarbose against α-glucosidase. Furthermore, the interaction between the isolated compounds and α-glucosidase was assessed by molecular docking. The results show that compound **6** exhibited the highest affinity to α-glucosidase.

Tyrosinase is the key enzyme that regulates the biosynthesis of melanin. Recently, the potential application of tyrosinase inhibitors to improve food quality and prevent pigmentation disorders has become important [44,45]. In the antityrosinase assay, the acetone extract of *Morus alba* L. exhibited stronger tyrosinase inhibitory activity than the positive control, arbutin. Furthermore, only compound **3** showed more potent antityrosinase activity than arbutin. The results indicate that compound **3** was approximately 100 times more effective against tyrosinase than arbutin. The interaction was further assessed by molecular docking of the isolated compounds and tyrosinase. The results show that compound **3** exhibited the highest affinity to tyrosinase.

Inflammation is an immune system response to defend against harmful stimuli, infection, and cellular damage. The induction of macrophages by LPS leads to the activation of TLR4 receptors, resulting in increased expression of iNOS and production of inflammatory factors, such as NO [46,47]. Our study results show that compounds **1**–**6** (especially 2) have considerable potential to inhibit NO production and iNOS expression, consistent with their high binding affinity to iNOS in molecular model docking and making them worthy of further investigation in the future.

## 5. Conclusions

Solvent extracts of *Morus alba* and their isolated compounds were tested using antioxidant systems, as well as anti-α-glucosidase, antityrosinase, and anti-inflammatory activity assays. Acetone extract of *Morus alba* showed significant antioxidant activities in DPPH, ABTS, superoxide radical scavenging, and FRAP assays. Acetone extract was found to have the highest TPC among all solvent extracts, which is consistent with its antioxidant activity results. Acetone extract also showed the strongest α-glucosidase and tyrosinase inhibitory properties. In terms of anti-inflammatory effect, ethyl acetate extract showed the highest anti-NO activity compared to other solvent extracts.

Six compounds isolated from *Morus alba* were quantified by HPLC and identified as morin (**1**), morusin (**2**), oxyresveratrol (**3**), umbelliferone (**4**), kuwanon G (**5**), and kuwanon H (**6**). Bioactivity tests showed that compounds **1** and **3** exhibited antioxidant activities in DPPH, ABTS radical scavenging, and FRAP assays. Furthermore, compounds **3** and **6** showed the strongest antityrosinase and anti-*α*-glucosidase properties, respectively, among all isolated compounds. As a result of molecular model docking, compounds **3** and **6** also showed the greatest binding affinity for tyrosinase and *α*-glucosidase, respectively, which is consistent with their potential antityrosinase and anti-*α*-glucosidase effects. Compound **2** showed the strongest anti-NO activity, which is consistent with its anti-iNOS effect in a Western blot assay and binding affinity to iNOS in molecular model docking.

In conclusion, in this study, we conducted a comparative assessment of the antioxidant, anti-α-glucosidase, antityrosinase, and anti-inflammatory activities of various solvent extracts and bioactive compounds from the root bark of *Morus alba* for the first time. As we hypothesized, these results are sufficient to demonstrate the importance of suitable solvents for extraction of bioactive compounds. The aforementioned bioactive extracts and their isolated compounds are useful as natural antioxidants in dietary supplements and in the food industry to prevent oxidative damage. In addition, acetone extract and compounds **3** and **6** should be further investigated as natural antityrosinase (especially **3**) and anti-α-glucosidase (especially **6**) agents for the treatment or prevention of pigmentation disorders and hyperglycemia. Ethyl acetate extract and compound **2** can be used as effective natural inflammation inhibitors to improve inflammation-related diseases, making them worthy of further research in the future.

## Figures and Tables

**Table 1:** TPC, TFC, and extraction yields of *Morus alba* L. with each extraction solvent.

Extracting Solvent	Relative Polarity	TPC (mg/g) ^a^ (GAE)	TFC (mg/g) ^b^ (QE)	Yield (%) ^c^
*n*-Hexane	0.009	0.00	99.58 ± 2.65 ***	1.5 ± 0.49
Dichloromethane	0.269	13.63 ± 0.33 ***	114.36 ± 4.94 ***	1.6 ± 0.61
Ethyl acetate	0.288	68.65 ± 6.12 **	156.20 ± 1.75 ***	1.9 ± 0.35
Acetone	0.355	94.61 ± 2.36 ***	167.22 ± 1.26 ***	2.0 ± 0.37
Ethanol	0.654	45.34 ± 0.77 ***	117.77 ± 2.01 ***	3.6 ± 0.84
Methanol	0.762	60.76 ± 1.41 ***	54.12 ± 1.37 ***	5.3 ± 1.19
Water	1.000	5.17 ± 1.48 *	20.73 ± 1.63 *	23.9 ± 5.50

^a^ TPC expressed in mg of gallic acid equivalents (GAE) per gram of extract; ^b^ TFC expressed in mg of quercetin equivalents (QE) per gram of extract; ^c^ yield was calculated as % yield = (weight of extract/initial weight of dry sample) × 100; values are expressed as means ± SD (*n* = 3); * *p* < 0.05, ** *p* < 0.01, *** *p* < 0.001 compared with the control.

**Table 2:** The antioxidant activities of various solvent extracts from *Morus alba* L. determined by DPPH, ABTS, superoxide radical scavenging, and FRAP assays.

Extracting Solvent	SC_50_ (μg/mL) ^a^			TE (mM/g) ^c^
	DPPH	ABTS	Superoxide	FRAP
*n*-Hexane	>400	>400	>400	50.83 ± 0.91 ***
Dichloromethane	>400	>400	>400	98.39 ± 0.69 ***
Ethyl acetate	>400	159.16 ± 10.74 **	388.74 ± 10.26 **	197.31 ± 2.11 ***
Acetone	242.33 ± 15.78 **	129.28 ± 10.53 **	124.80 ± 22.09 **	213.67 ± 2.06 ***
Ethanol	331.81 ± 22.55 **	118.25 ± 3.29 **	>400	181.83 ± 3.24 ***
Methanol	308.10 ± 8.24 *	65.60 ± 6.03 **	299.13 ± 26.45 *	200.51 ± 2.53 ***
Water	>400	257.38 ± 32.53 *	>400	52.32 ± 1.91 ***
BHT ^b^	19.75 ± 1.79 *	25.87 ± 5.83 *	N.A. ^d^	4385.56 ± 45.54 ***

^a^ SC_50_ value defined as the concentration of the samples causing 50% free radical scavenging and displayed as mean ± SD (*n* = 3); ^b^ butylated hydroxytoluene (BHT) used as positive control; ^c^ FRAP expressed in mM of Trolox equivalents (TE) per gram of extract; ^d^ N.A., not available; * *p* < 0.05, ** *p* < 0.01, and *** *p* < 0.001 compared with the control.

**Table 3:** *α*-Glucosidase and tyrosinase inhibitory activities of various solvent extracts.

Compound	IC_50_ (μg/mL) ^a^	
	*α*-Glucosidase	Tyrosinase
*n*-Hexane	22.85 ± 4.51 *	>400
Dichloromethane	12.04 ± 6.67 **	>400
Ethyl acetate	5.80 ± 2.29 **	11.27 ± 2.75 **
Acetone	3.87 ± 1.95 ***	7.95 ± 1.54 **
Ethanol	11.48 ± 1.81 *	19.86 ± 3.35 **
Methanol	12.48 ± 0.93 **	13.41 ± 0.77 ***
Water	17.94 ± 6.37 **	>400
Acarbose ^b^	355.48 ± 28.39 *	-
Arbutin ^b^	-	53.51 ± 8.87 *

^a^ IC_50_ value defined as half-maximal inhibitory concentration and expressed as mean ± SD (*n* = 3); ^b^ acarbose and arbutin were used as positive controls; * *p* < 0.05, ** *p* < 0.01, and *** *p* < 0.001 compared with the control.

**Table 4:** The effects of solvent extracts on nitric oxide (NO) production in RAW 264.7 cells.

Compound	NO Inhibition IC_50_ (μg/mL) ^a^
*n*-Hexane	53.22 ± 17.28 *
Dichloromethane	15.33 ± 2.63 *
Ethyl acetate	10.81 ± 1.41 *
Acetone	12.00 ± 1.32 *
Ethanol	14.30 ± 2.38 *
Methanol	17.19 ± 2.04 *
Water	121.08 ± 7.85 *
Quercetin ^b^	3.98 ± 0.28 *

^a^ IC_50_ value defined as half-maximal inhibitory concentration and expressed as mean ± SD (*n* = 3); ^b^ quercetin was used as a positive control; * *p* < 0.05 compared with the control.

**Table 5:** Identification and quantification of the active components of *Morus alba* L. in solvent extracts.

Extracting Solvent	Morin (mg/g)	Morusin (mg/g)	Oxyresveratrol (mg/g)	Umbelliferone (mg/g)	Kuwanon G (mg/g)	Kuwanon H (mg/g)	Total Amount (mg/g)
*n*-Hexane	2.23 ± 0.18	3.92 ± 0.25	0.64 ± 0.08	2.35 ± 0.16	11.27 ± 0.92	2.46 ± 0.18	22.87 ± 1.77
Dichloromethane	0.92 ± 0.10	9.96 ± 0.98	1.22 ± 0.13	2.93 ± 0.18	8.24 ± 0.88	5.43 ± 0.58	28.70 ± 4.31
Ethyl acetate	2.56 ± 0.42	7.81 ± 0.62	2.99 ± 0.19	3.29 ± 0.12	12.84 ± 1.02	8.32 ± 0.79	37.81 ± 3.16
Acetone	3.24 ± 0.26	8.64 ± 0.92	3.33 ± 0.22	2.23 ± 0.12	18.54 ± 1.82	21.24 ± 1.94	57.22 ± 5.28
Ethanol	3.51 ± 0.27	5.82 ± 0.47	2.65 ± 0.21	1.43 ± 0.07	19.04 ± 1.76	26.74 ± 2.24	59.19 ± 5.02
Methanol	1.60 ± 0.09	4.14 ± 0.36	3.17 ± 0.28	2.73 ± 0.13	22.64 ± 2.02	29.34 ± 2.83	63.62 ± 5.71
Water	2.87 ± 0.24	4.07 ± 0.48	2.41 ± 0.22	1.22 ± 0.18	18.38 ± 1.24	N.D.	28.95 ± 2.36

Results are expressed as micrograms of each compound per gram of extract.

**Table 6:** Antioxidant activities of isolated components from *Morus alba* L. determined by DPPH, ABTS, superoxide radical scavenging, and FRAP assays.

Compound	SC_50_ (μM) ^a^			TE (mM/g) ^c^
	DPPH	ABTS	Superoxide	FRAP
Morin (**1**)	17.49 ± 3.43 *	12.67 ± 5.98 ***	54.50 ± 18.96 *	2963.28 ± 63.65 ***
Morusin (**2**)	>200	44.19 ± 8.07 ***	>200	583.43 ± 25.79 ***
Oxyresveratrol (**3**)	44.42 ± 12.51 *	9.50 ± 2.08 ***	>200	2402.22 ± 32.91 ***
Umbelliferone (**4**)	>200	>200	>200	145.23 ± 35.46 **
Kuwanon G (**5**)	>200	9.28 ± 1.02 **	188.24 ± 19.07 *	3549.22 ± 160.65 *
Kuwanon H (**6**)	>200	8.68 ± 0.93 **	>200	1750.96 ± 36.30 **
BHT ^b^	89.84 ± 7.03 **	115.86 ± 25.14 *	N.A. ^d^	4385.56 ± 78.88 ***

^a^ SC_50_ value defined as the concentration of the samples causing 50% free radical scavenging and displayed as mean ± SD (*n* = 3); ^b^ butylated hydroxytoluene (BHT) used as positive control; ^c^ FRAP expressed in mM of Trolox equivalents (TE) per gram of extract; ^d^ N.A., not available; * *p* < 0.05, ** *p* < 0.01, and *** *p* < 0.001 compared with the control.

**Table 7:** *α*-Glucosidase and tyrosinase inhibitory activities of isolated compounds.

Compound	IC_50_ (μM) ^a^	
	*α*-Glucosidase	Tyrosinase
Morin (**1**)	90.15 ± 10.17 *	199.41 ± 84.44 **
Morusin (**2**)	15.66 ± 2.15 **	>400
Oxyresveratrol (**3**)	8.35 ± 0.46 *	1.89 ± 0.59 **
Umbelliferone (**4**)	492.46 ± 12.82 *	>400
Kuwanon G (**5**)	7.00 ± 0.31 *	100.40 ± 9.62 *
Kuwanon H (**6**)	4.06 ± 0.12 *	218.00 ± 40.96 *
Acarbose ^b^	540.38 ± 47.42 *	-
Arbutin ^b^	-	206.08 ± 36.50 *

^a^ IC_50_ value defined as half-maximal inhibitory concentration and expressed as mean ± SD (*n* = 3); ^b^ acarbose and arbutin were used as positive controls; * *p* < 0.05 and ** *p* < 0.01 compared with the control.

**Table 8:** Effects of the isolated components on nitric oxide (NO) production in RAW 264.7 cells.

Compound	NO Inhibition IC_50_ (μM) ^a^
Morin (**1**)	43.26 ± 4.61 *
Morusin (**2**)	9.87 ± 0.59 *
Oxyresveratrol (**3**)	25.36 ± 3.47 *
Umbelliferone (**4**)	36.76 ± 2.26 *
Kuwanon G (**5**)	17.80 ± 0.50 *
Kuwanon H (**6**)	40.48 ± 4.38 *
Quercetin ^b^	13.20 ± 2.00 *

^a^ IC_50_ value defined as half-maximal inhibitory concentration and expressed as mean ± SD (*n* = 3); ^b^ quercetin was used as a positive control; * *p* < 0.05 compared with the control.

**Table 9:** Binding energies of active components and acarbose calculated in silico.

Compound	Affinity (kcal/mol)
Morin (**1**)	−7.6
Morusin (**2**)	−8.0
Oxyresveratrol (**3**)	−8.2
Umbelliferone (**4**)	−5.9
Kuwanon G (**5**)	−8.5
Kuwanon H (**6**)	−9.9
Acarbose ^a^	−5.6

^a^ Acarbose used as a positive control.

**Table 10:** Binding energies of bioactive compounds and arbutin calculated in silico.

Compound	Affinity (kcal/mol)
Morin (**1**)	−6.1
Morusin (**2**)	−5.5
Oxyresveratrol (**3**)	−7.3
Umbelliferone (**4**)	−5.3
Kuwanon G (**5**)	−6.4
Kuwanon H (**6**)	−5.9
Arbutin ^a^	−6.1

^a^ Arbutin used as a positive control.

**Table 11:** Binding energies of inducible nitric oxide synthase (iNOS) from *Mus musculus* in silico.

Compound	Affinity (kcal/mol)
Morin (**1**)	−7.0
Morusin (**2**)	−9.7
Oxyresveratrol (**3**)	−7.5
Umbelliferone (**4**)	−6.9
Kuwanon G (**5**)	−8.1
Kuwanon H (**6**)	−7.3
Quercetin ^a^	−7.9

^a^ Quercetin used as positive control.

## Data Availability

Data are contained within the article.

## Supplementary Materials

The following supporting information can be downloaded at: https://www.mdpi.com/article/10.3390/antiox11112222/s1. Table S1: Retention time, LODs, LOQs, and regression analyses for six components of *Morus alba* L. in reverse-phase HPLC. Figure S1: The ^1^H-NMR spectrum of morin (**1**). Figure S2: The ^1^H-NMR spectrum of morusin (**2**). Figure S3: The ^1^H-NMR spectrum of oxyresveratrol (**3**). Figure S4: The ^1^H-NMR spectrum of umbelliferone (**4**). Figure S5: The ^1^H-NMR spectrum of kuwanon G (**5**). Figure S6: The ^1^H-NMR spectrum of kuwanon H (**6**). Figure S7: HPLC chromatogram of isolated pure compounds in reverse phase. Figure S8: Reverse-phase HPLC chromatogram of water extract. Figure S9: Reverse-phase HPLC chromatogram of methanol extract. Figure S10: Reverse-phase HPLC chromatogram of ethanol extract. Figure S11: Reverse-phase HPLC chromatogram of acetone extract. Figure S12: Reverse-phase HPLC chromatogram of ethyl acetate extract. Figure S13: Reverse-phase HPLC chromatogram of dichloromethane extract. Figure S14: Reverse-phase HPLC chromatogram of *n*-hexane extract.
