# Supplementary material for: Antioxidant, Anti-α-Glucosidase, Antityrosinase, and Anti-Inflammatory Activities of Bioactive Components from Morus alba
Source: Antioxidants (Basel). 2022 Nov 11;11(11):2222. doi: 10.3390/antiox11112222 (PMC9686747; doi:10.3390/antiox11112222)
Supplement: Supplementary file 1 [file antioxidants-11-02222-s001.zip › antioxidants-2025351-supplementary.pdf]

Supplementary data

Antioxidant, Anti-α-Glucosidase, Anti-Tyrosinase, and Anti-Inflammatory Activities of Bioactive Components from *Morus alba*

Jui-Hung Hsu [1], Chang-Syun Yang [1] and Jih-Jung Chen [1,2,*]

[1] Department of Pharmacy, School of Pharmaceutical Sciences, National Yang Ming Chiao Tung University, Taipei 112, Taiwan

[2] Department of Medical Research, China Medical University Hospital, China Medical University, Taichung 404332, Taiwan

* Correspondence: jjungchen@nycu.edu.tw; Tel.: +886-2-2826-7195; Fax: +886-2-2823-2940

Contents

Table S1. Retention time, LODs, LOQs, and regression analyses for six components of *Morus alba L.* in reversed-phase HPLC..S3

Figure S1. The ^{1}H-NMR spectrum (600 MHz, $CDCl_3$) of morin (**1**) S4

Figure S2. The ^{1}H-NMR spectrum (600 MHz, $CDCl_3$) of morusin (**2**) S4

Figure S3. The ^{1}H-NMR spectrum (600 MHz, methanol-d_4) of oxyresveratrol (**3**) S5

Figure S4. The ^{1}H-NMR spectrum (600 MHz, $CDCl_3$) of umbelliferone (**4**) S5

Figure S5. The ^{1}H-NMR spectrum (600 MHz, methanol-d_4) of kuwanon G (**5**) S6

Figure S6. The ^{1}H-NMR spectrum (600 MHz, methanol-d_4) of kuwanon H (**6**) S6

Figure S7. Reversed-phase HPLC chromatogram of isolated pure compounds......................S7

Figure S8. Reversed-phase HPLC chromatogram of water extract...S7

Figure S9. Reversed-phase HPLC chromatogram of methanol extract.................................... S8

Figure S10. Reversed-phase HPLC chromatogram of ethanol extract.......................................S8

Figure S11. Reversed-phase HPLC chromatogram of acetone extract.......................................S8

Figure S12. Reversed-phase HPLC chromatogram of ethyl acetate extract..............................S9

Figure S13. Reversed-phase HPLC chromatogram of dichloromethane extract......................S9

Figure S14. Reversed-phase HPLC chromatogram of *n*-hexane extract…..............................S9

Table S1. Retention time, LODs, LOQs, and regression analyses for six components of *Morus alba L.* in reversed-phase HPLC.

Compounds	T_m (min) [a]	Regression equation	Correlation coefficient	LOD (μg/mL) [a]	LOQ (μg/mL) [a]
Umbelliferone	19.0	y = 0.048x + 0.003	0.9998	3.30	10.00
Oxyresveratrol	20.0	y = 2.826x + 0.006	0.9986	0.67	2.05
Morin	23.0	y = 0.107x - 0.034	0.9992	14.18	44.86
Kuwanon G	36.0	y = 0.490x + 0.018	0.9982	3.64	11.02
Kuwanon H	41.0	y = 0.162x + 0.013	0.9986	12.83	38.89
Morusin	50.8	y = 0.062x + 0.006	0.9990	3.03	9.19

[a] T_m: Retention time; LOD: Limit of detection; LOQ : Limit of quantification

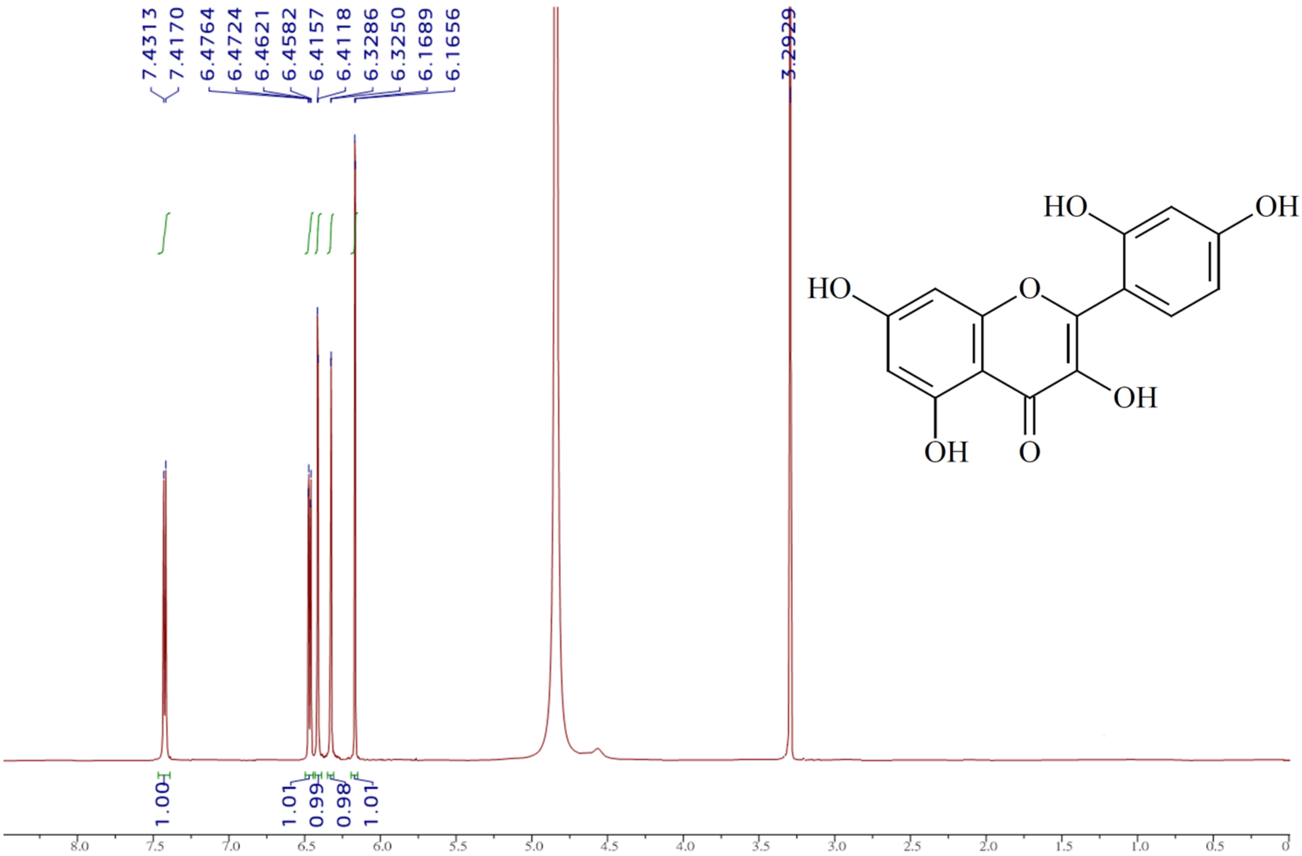

Figure S1. The ^{1}H-NMR spectrum (600 MHz, $CDCl_3$) of morin (**1**).

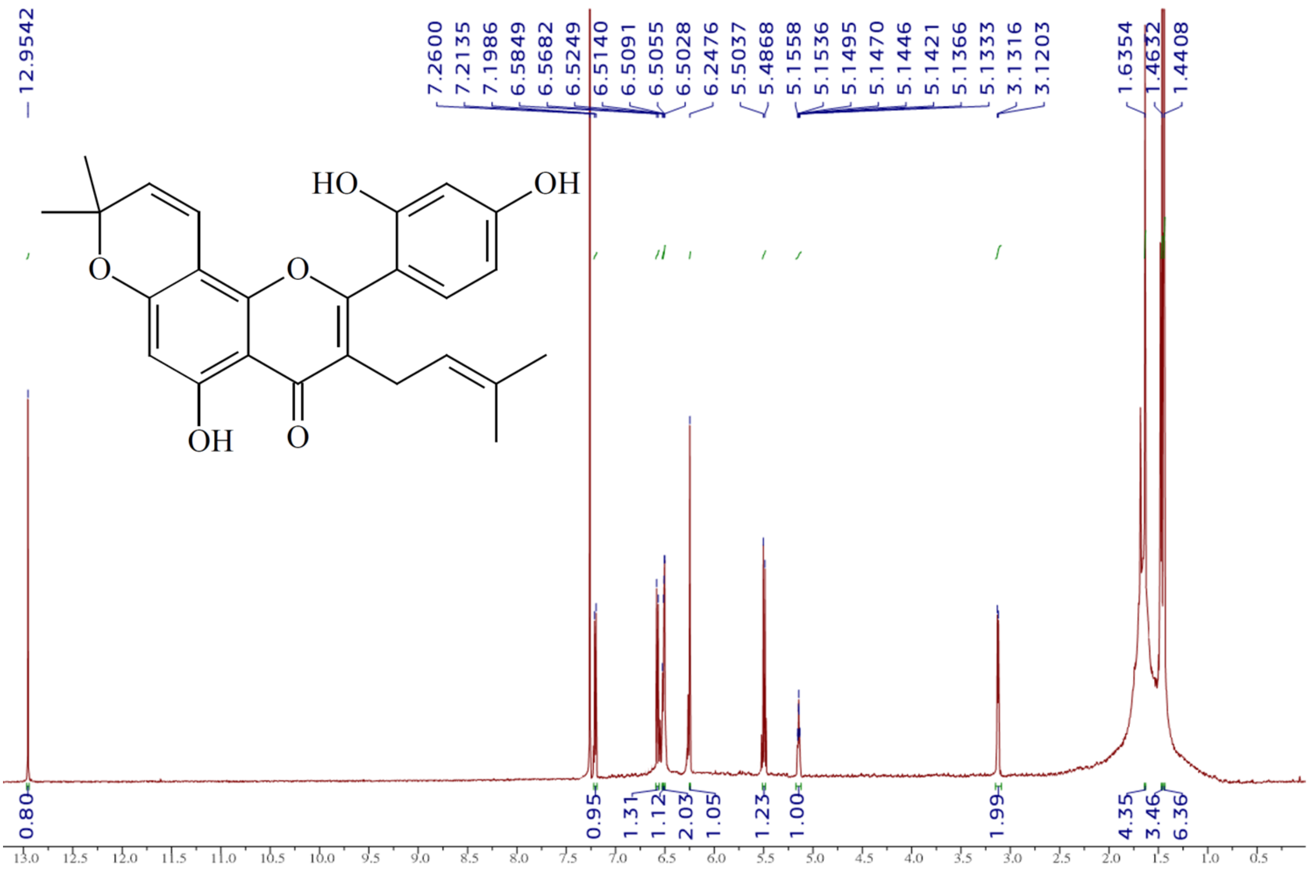

Figure S2. The ^{1}H-NMR spectrum (600 MHz, $CDCl_3$) of morusin (**2**).

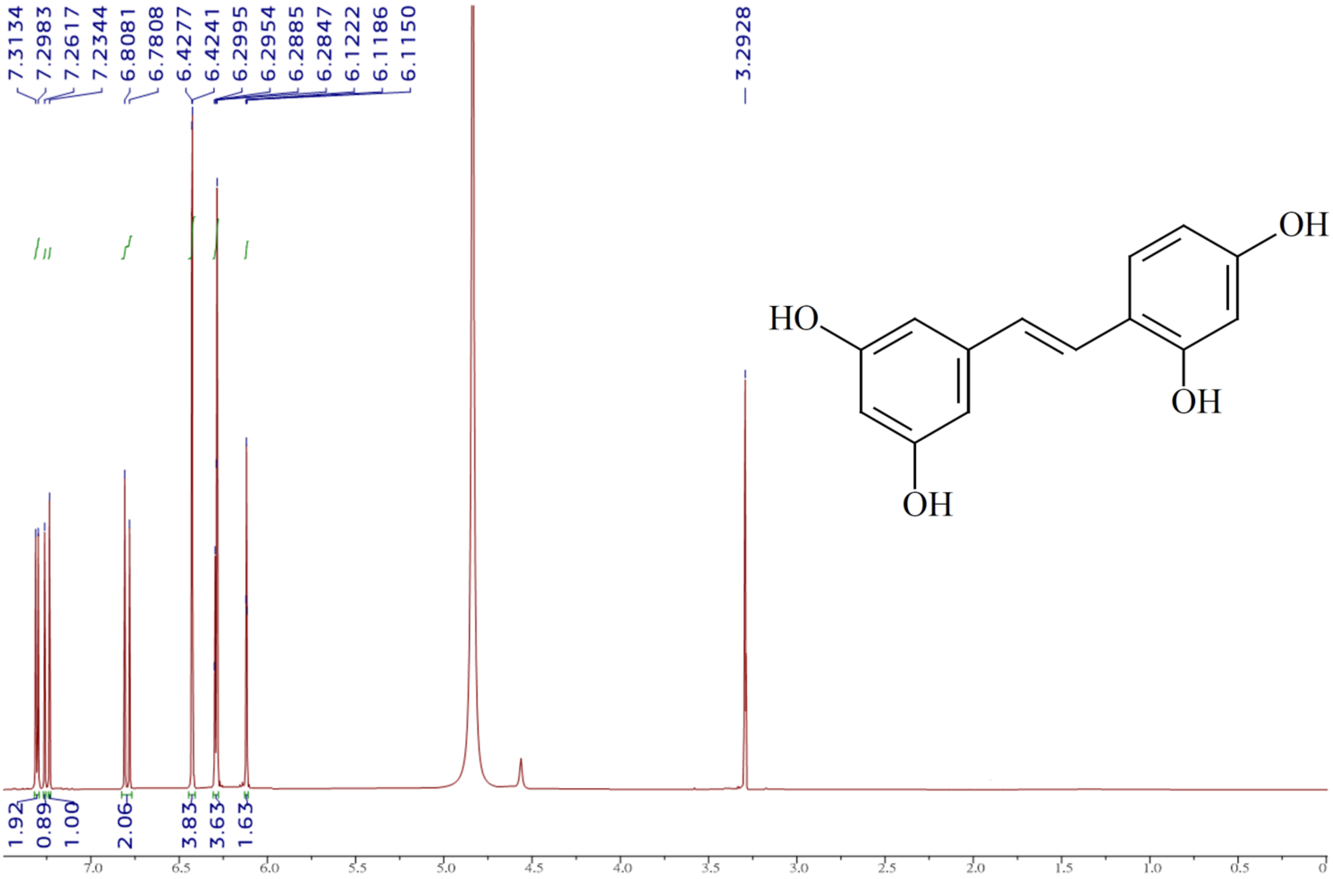

Figure S3. The ^{1}H-NMR spectrum (600 MHz, methanol-d_4) of oxyresveratrol (**3**).

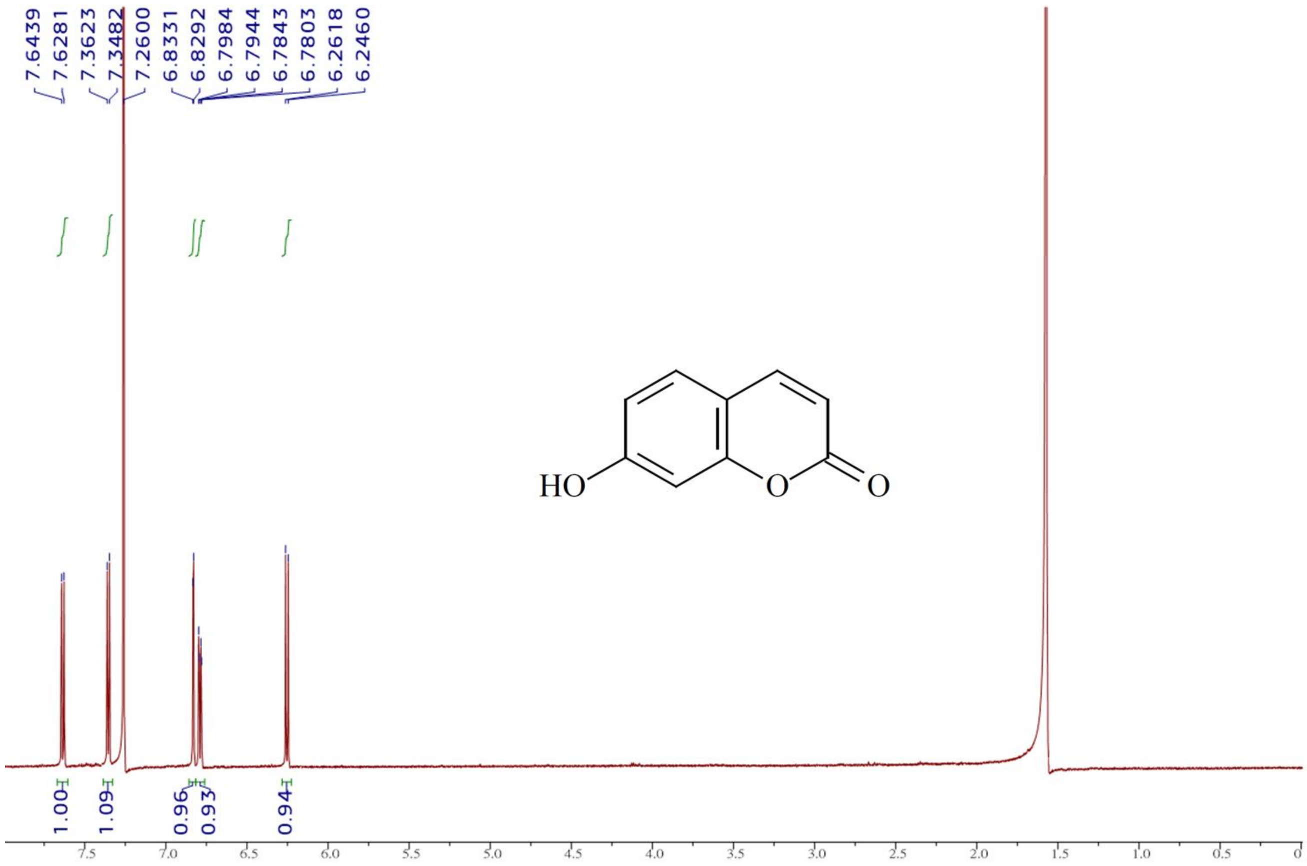

Figure S4. The ^{1}H-NMR spectrum (600 MHz, $CDCl_3$) of umbelliferone (**4**).

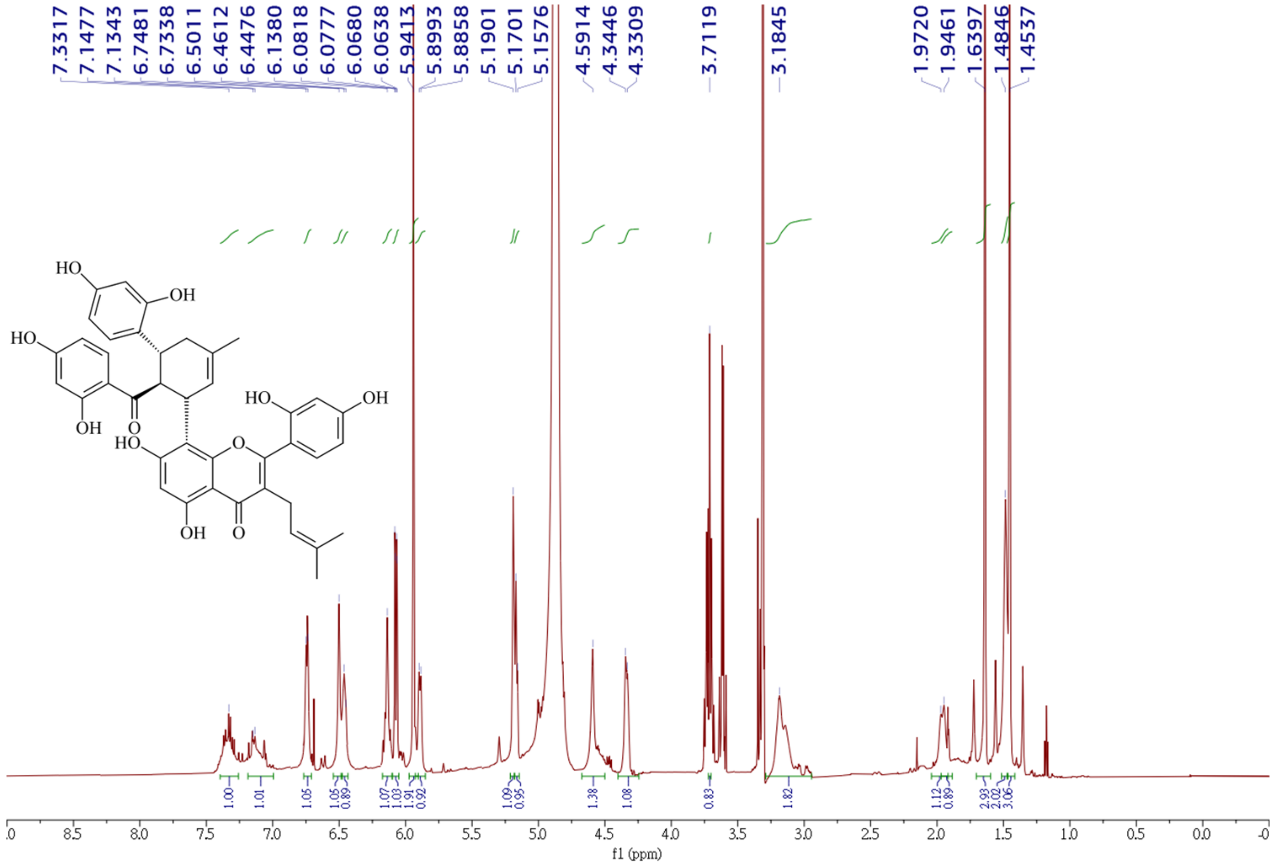

Figure S5. The ^{1}H-NMR spectrum (600 MHz, methanol-d_4) of kuwanon G (**5**).

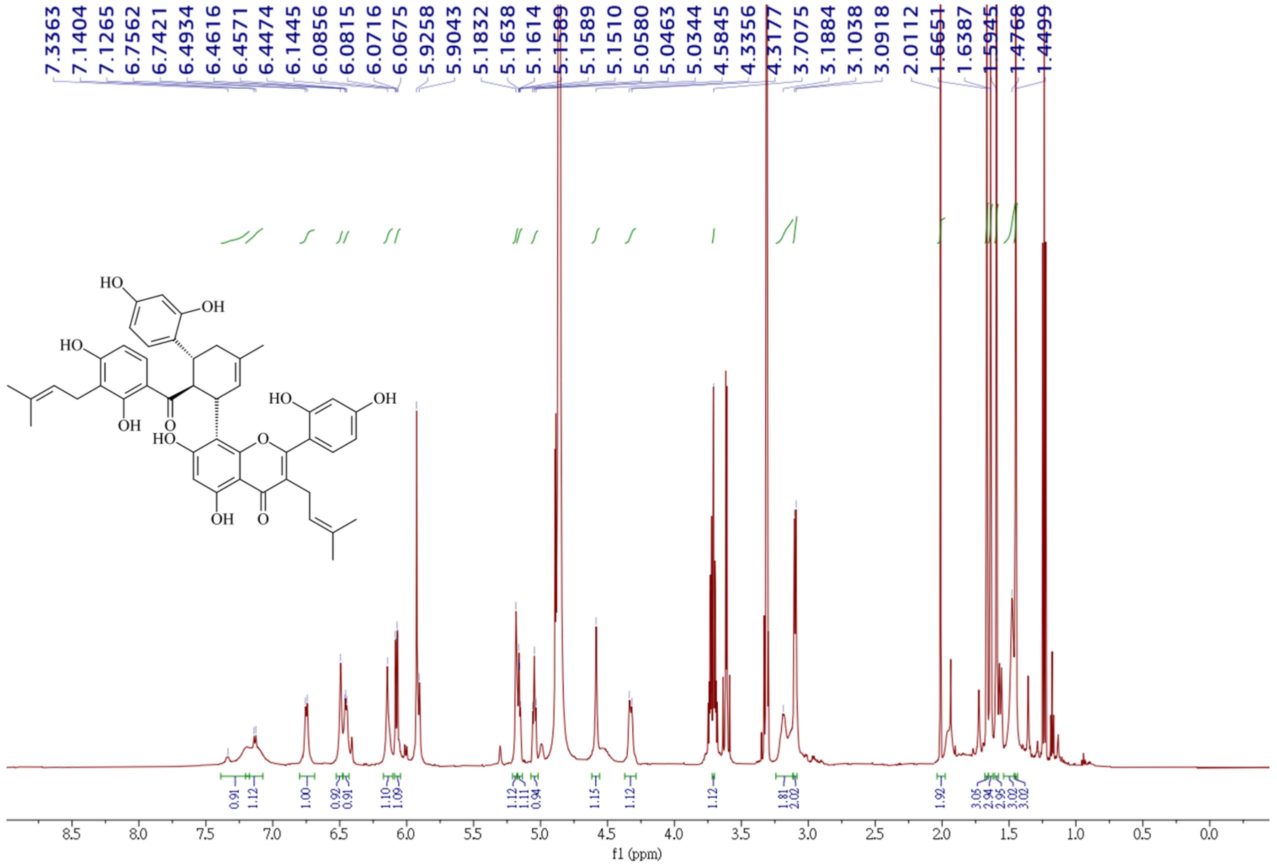

Figure S6. The ^{1}H-NMR spectrum (600 MHz, methanol-d_4) of kuwanon H (**6**).

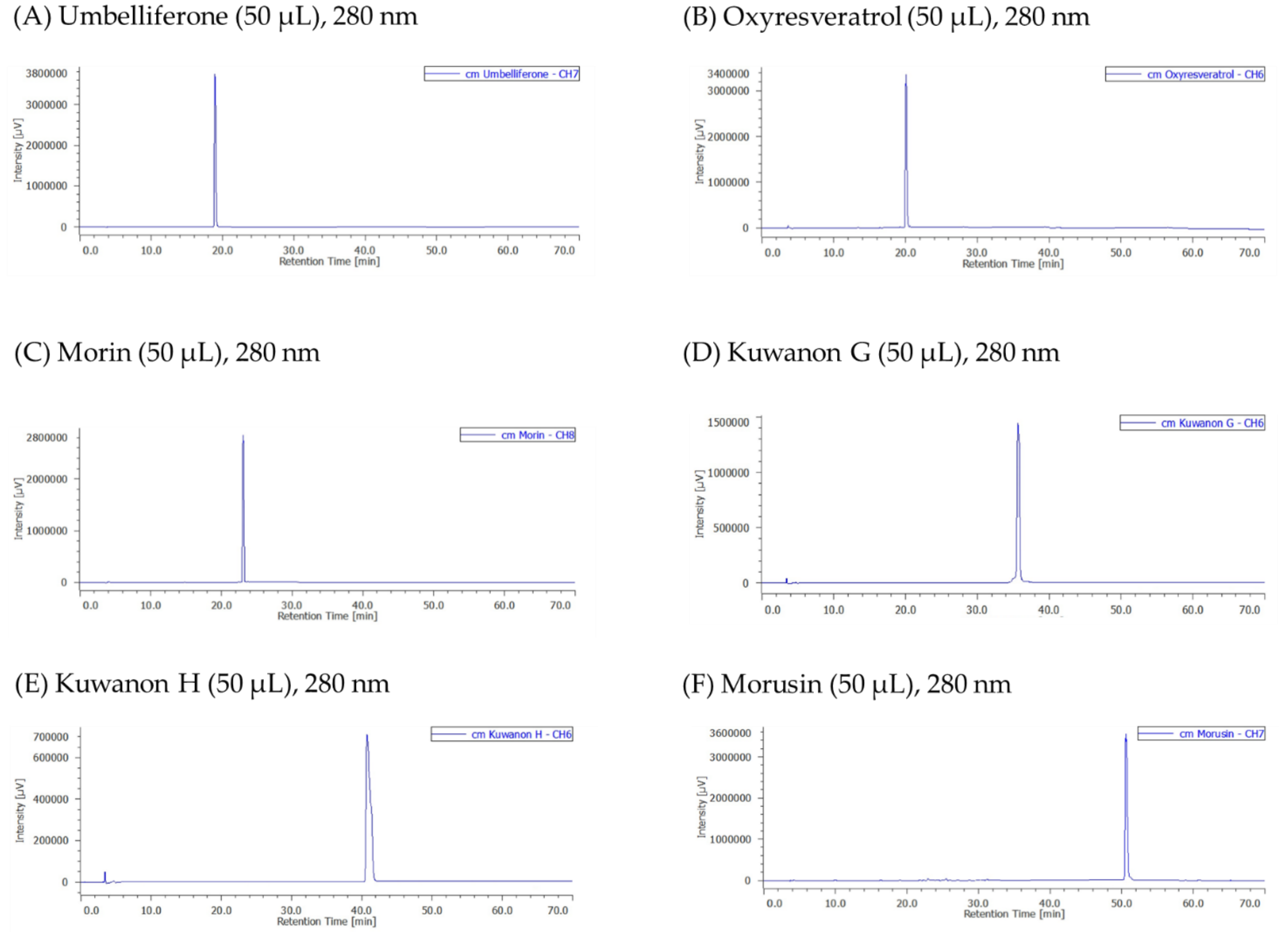

Figure S7. Reversed-phase HPLC chromatogram of isolated pure compounds (A to F).

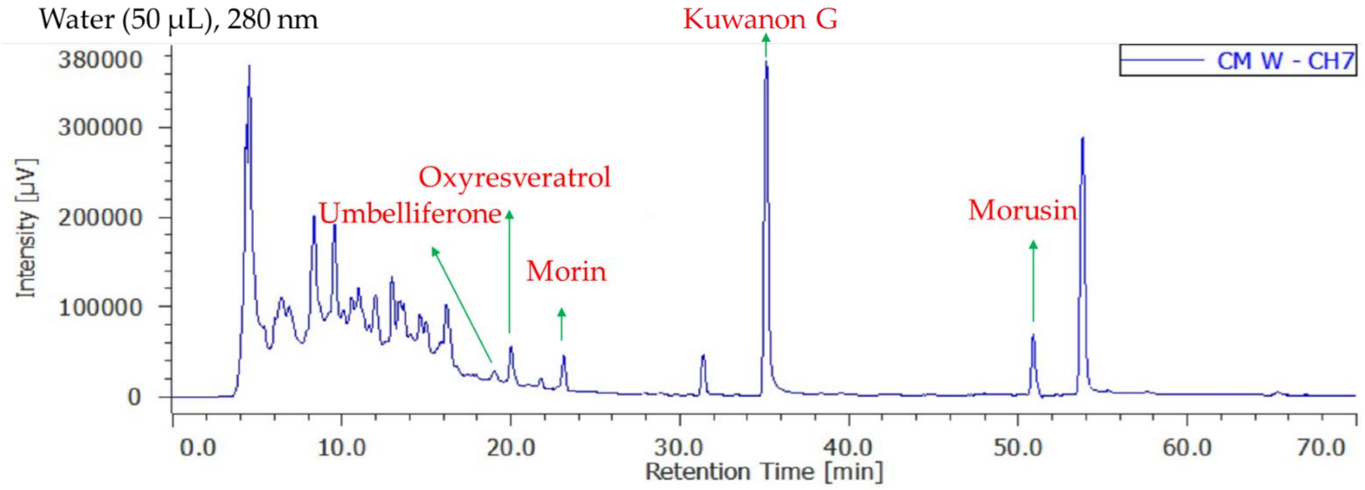

Figure S8. Reversed-phase HPLC chromatogram of water extract.

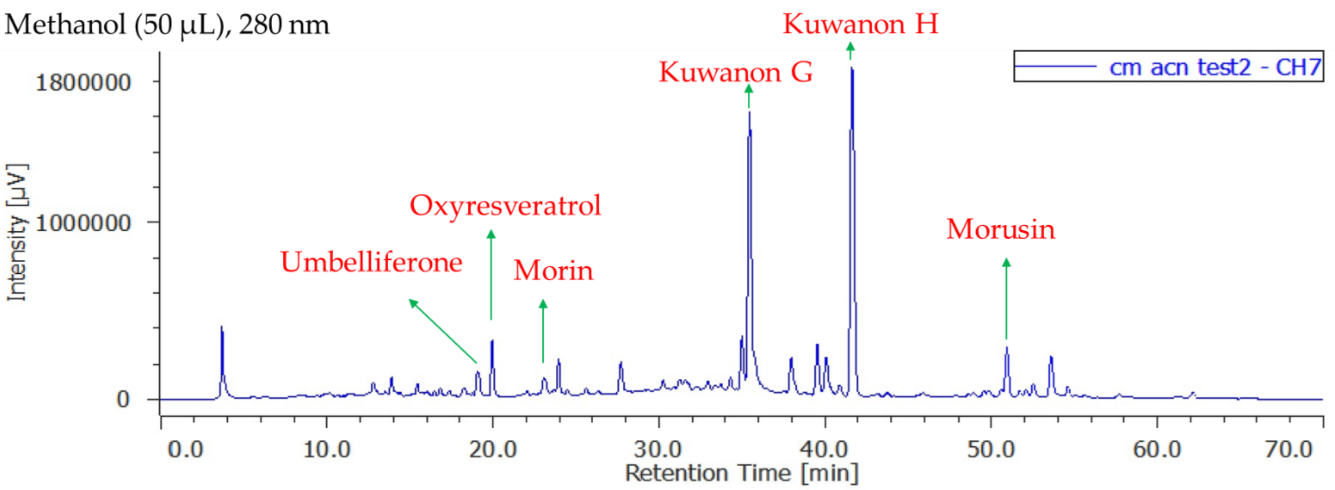

Figure S9. Reversed-phase HPLC chromatogram of methanol extract.

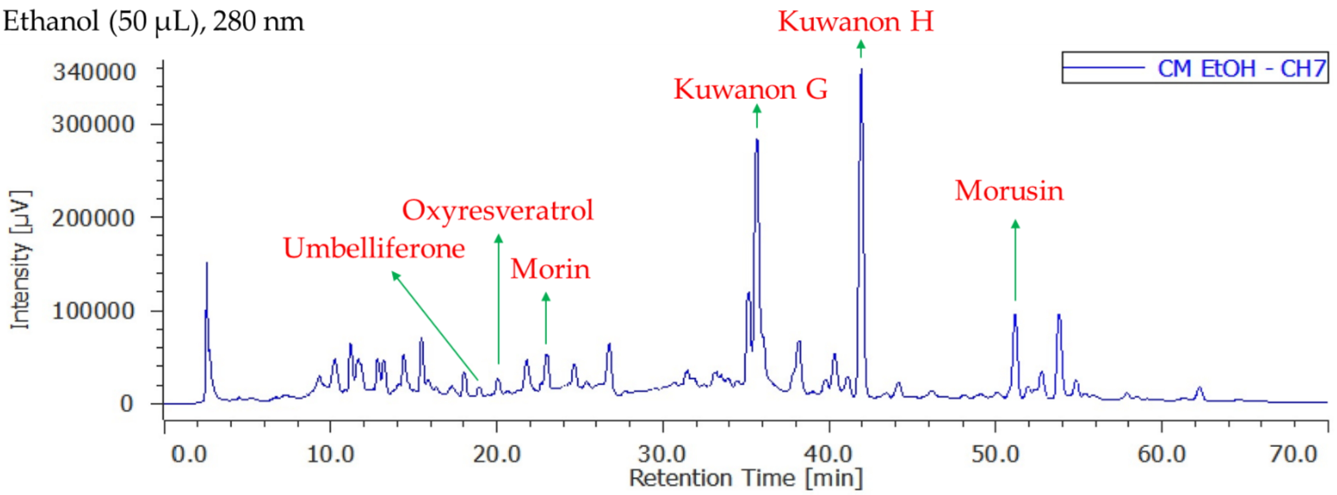

Figure S10. Reversed-phase HPLC chromatogram of ethanol extract.

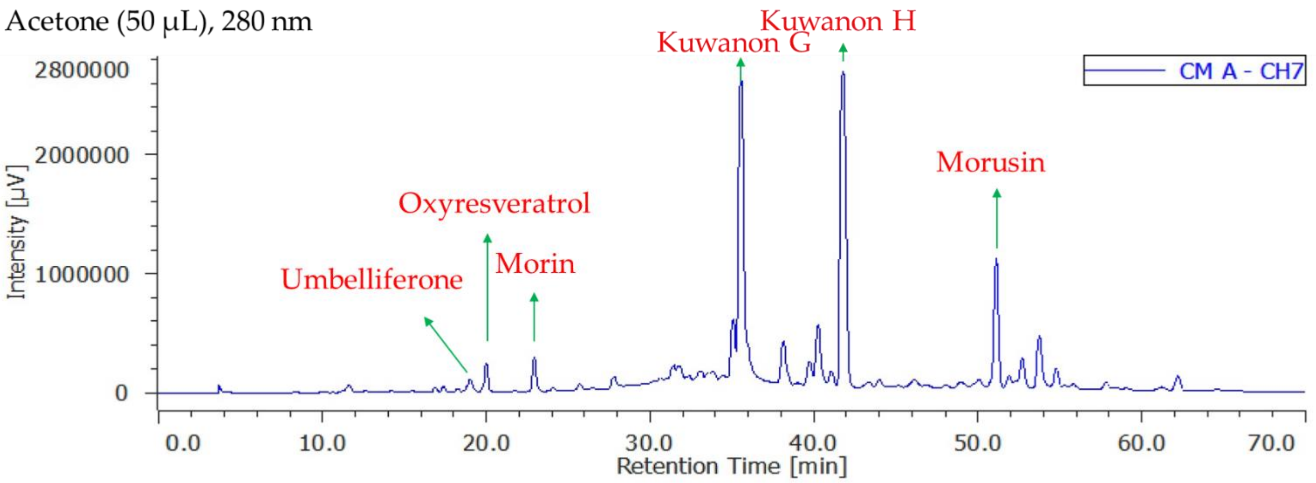

Figure S11. Reversed-phase HPLC chromatogram of acetone extract.

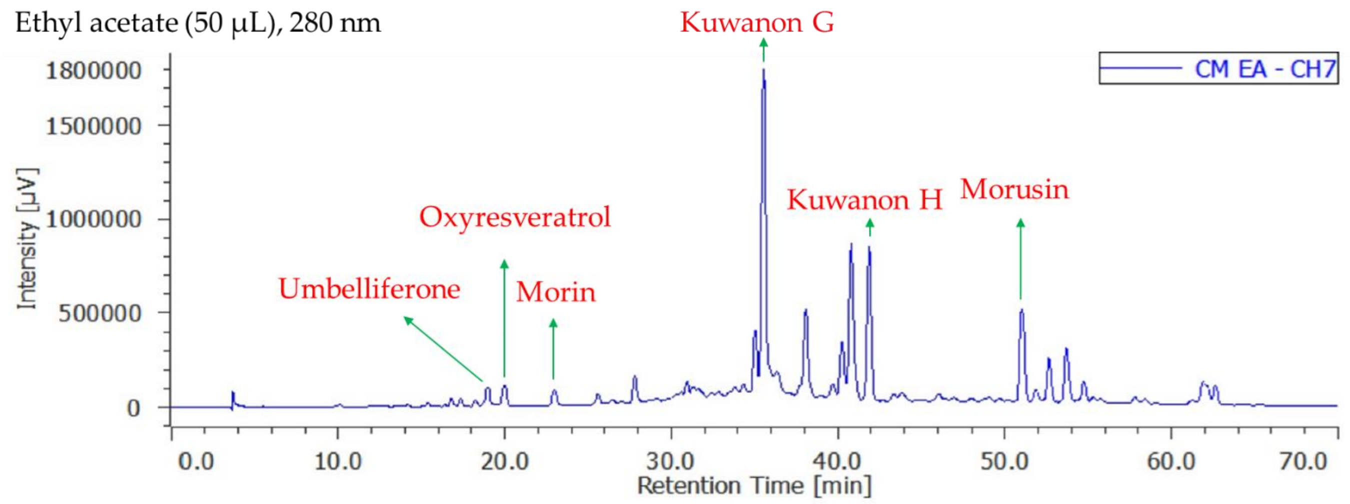

Figure S12. Reversed-phase HPLC chromatogram of ethyl acetate extract.

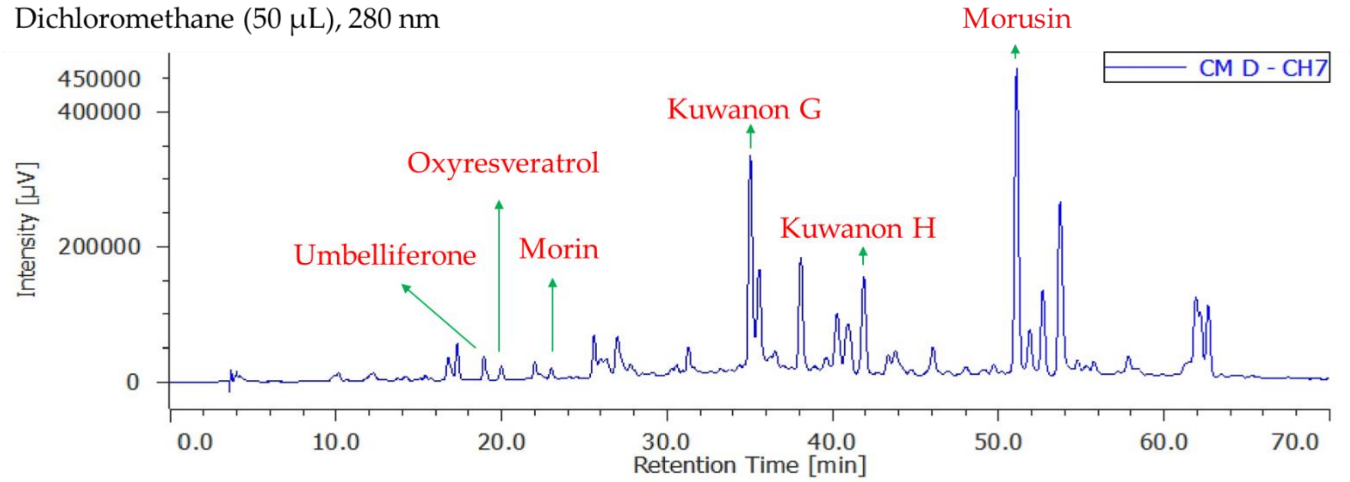

Figure S13. Reversed-phase HPLC chromatogram of dichloromethane extract.

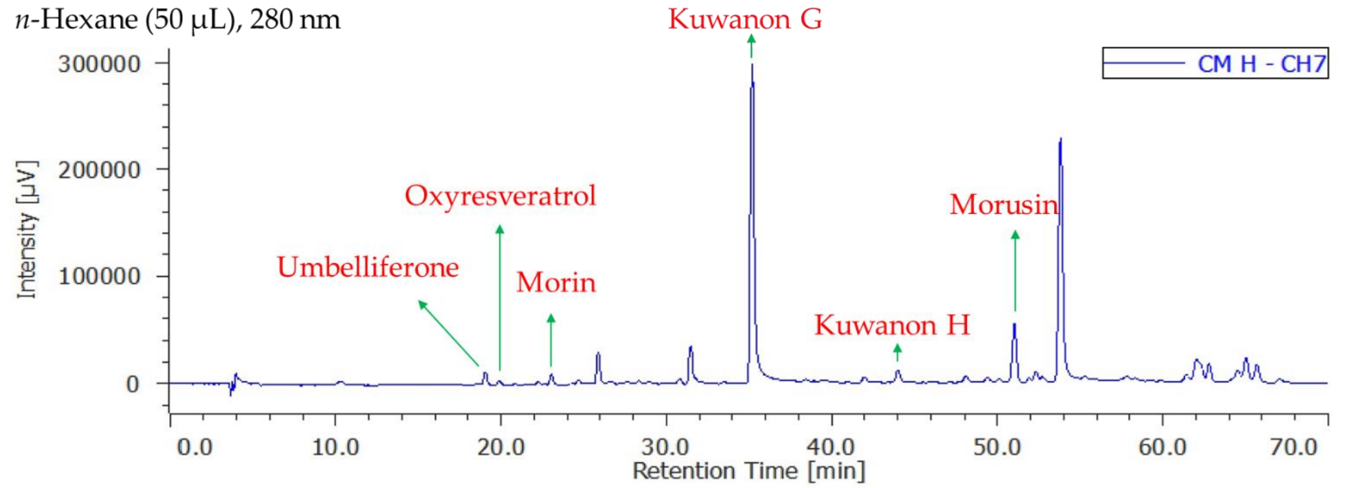

Figure S14. Reversed-phase HPLC chromatogram of *n*-hexane extract.
